# Development of a competency framework for public health nurses in China: A modified Delphi study

**DOI:** 10.1371/journal.pone.0361940

**Published:** 2026-10-30

**Authors:** Chunxia He, Lingli Yan, Jia Yin, Qian Yao, Jiali Zhang, Jimei Zhang, Xiuying Hu

**Affiliations:** 1 Emergency Department, The Third People's Hospital of Chengdu, Chengdu, China; 2 Innovation Center of Nursing Research, West China Hospital, Sichuan University, Chengdu, China; 3 Nursing Key Laboratory of Sichuan Province, West China Hospital, Sichuan University, Chengdu, China; 4 Nursing Department, Shang Jin Hospital of West China Hospital, Sichuan University, Chengdu, China; Universiti Sains Malaysia - Kampus Kesihatan, MALAYSIA

## Abstract

**Background:**

Public health nurses play a pivotal role in primary public health services, yet China lacks a systematic, context-specific competency framework to support workforce development. This study aimed to develop a competency framework for public health nurses in China to guide capacity-building initiatives.

**Methods:**

A preliminary competency framework was constructed based on a literature review, job analysis, and semi-structured interviews with 22 frontline practitioners and comprised six domains, 16 subdomains, and 52 competencies. A two-round Delphi survey was then conducted with 21 experts from multiple regions with expertise in community nursing, public health, primary care medicine, and nursing education and related specialties. Consensus was defined as a mean importance rating ≥4.0 (5-point Likert scale) and a coefficient of variation ≤0.25. The Delphi consultation process was characterized by expert engagement, the expert authority coefficient, and the coordination of expert opinions. Finally, the Analytic Hierarchy Process was applied to determine the relative weights of the agreed-upon items at each level.

**Results:**

Across the two Delphi rounds, the response rates were 95.45% and 100%, the expert authority coefficient was 0.90, and Kendall’s coefficients of concordance were 0.156 and 0.171, respectively (both P < 0.001). The final competency framework comprised six domains, 16 subdomains, and 46 competencies. All Analytic Hierarchy Process judgment matrices showed acceptable consistency (CR < 0.10). Health education and health promotion was assigned the highest weight (0.280) among the domains.

**Conclusion:**

The resulting competency framework demonstrates methodological rigor, a comprehensive structure, and contextual relevance. It provides theoretical and practical implications for competency-based education, workforce development, and the professionalization of public health nursing in China.

## Introduction

In recent years, the growing burden of non-communicable diseases (NCDs) [1] and recurrent outbreaks of emerging infectious diseases (EIDs) [2] have posed sustained threats to population health and global health security, placing increasing demands on the capacity and preparedness of the health workforce. In response, the World Health Organization (WHO), through its *Global Strategy on Human Resources for Health: Workforce 2030*, has emphasized the need to strengthen the health workforce to build resilient health systems [3]. Consistent with this strategy, China has prioritized the public health workforce through the *Healthy China 2030 Planning Outline* [4] and the *15th Five-Year Plan* [5].

Public health nurses (PHNs), as direct providers and key implementers of public health services, play an increasingly important role in addressing these health challenges. Internationally, PHNs constitute an established component of the public health workforce in many countries. Evidence demonstrated that public health nursing contributes to reducing disease risk [6], protecting and promoting population health [7], strengthening preparedness for and response to public health emergencies [8], and advancing national and global health goals [9]. In China, however, “public health nurse” is not an officially designated professional title. The term in this study refers to nurses working in primary health care institutions who undertake public health nursing responsibilities. These nurses may be referred to as “community public health nurses” or “public health department nurses” in practice. Their responsibilities primarily include disease prevention, health promotion, health education, chronic disease management, health management for key populations, and family planning services. PHNs constitute 40.8% of China’s primary-level public health workforce [10], and contribute to the delivery of all 55 service categories under the National Basic Public Health Services (BPHS) program. Despite these contributions, public health nursing in China remains at a nascent and exploratory stage and faces multiple practical challenges, including low levels of educational attainment and an imbalanced professional title structure among personnel [11], a lack of systematic public health nursing education [10], and suboptimal service efficiency [12,13]. There is an urgent need to strengthen the capacity of this workforce and to transition public health nursing services toward higher quality and greater efficiency.

Competency frameworks serve as important references for workforce development and provide a structured foundation for practice, education, and training by defining the competencies required for effective practice. Several countries, including the United States [14], Canada [15], and Japan [16], have developed relatively mature competency frameworks for PHNs. The content and structure of these competency frameworks are fundamentally shaped by the scope of public health nursing practice in their respective healthcare systems. In the United States, public health nursing practice is guided by the Minnesota Public Health Intervention Wheel [17], which adopts population-based practice as its core principle and encompasses interventions at the individual, community, and system levels. Japan adopts a concentric-circle approach [18] that conceptualizes public health nursing as an integrated continuum extending from health guidance for individuals and families to community health activities, policy development, and health system management. Despite differences in their organizational models, both approaches conceptualize public health nursing as a multilevel practice that extends from individuals and families to communities and health systems.

In contrast, public health nursing practice in China is primarily organized around the nationally standardized Basic Public Health Services (BPHS) program delivered through primary health care institutions. Practice is guided by standardized service specifications and focuses on the routine delivery of essential public health services. Although international frameworks provide valuable conceptual guidance, they require adaptation to the context of public health nursing practice in China rather than direct transfer. Furthermore, research on public health nursing competencies in China remains limited and fragmented, leaving insufficient evidence to guide the professionalization and standardization of the public health nursing workforce. Therefore, this study aimed to develop a competency framework for PHNs in China, grounded in current national realities and responsive to evolving trends in public health nursing practice. The framework may provide a structured reference for PHN workforce development and contribute to the professionalization of public health nursing in China.

## Methods

### Design

This study employed a modified Delphi design to systematically integrate expert opinions and achieve consensus, comprising two phases (Fig 1). In Phase 1, a preliminary competency framework was developed based on three sources of evidence: a literature review, a job analysis of national service standards, and semi-structured interviews with 22 frontline practitioners. This phase served as an alternative to the traditional open-ended first Delphi round. In Phase 2, the framework was refined through two rounds of structured expert consultation until the predefined consensus criteria were met. The study was reported in accordance with the Conducting and Reporting of Delphi Studies (CREDES) checklist [19], and the completed checklist was provided as S1 File.

**Fig 1:**
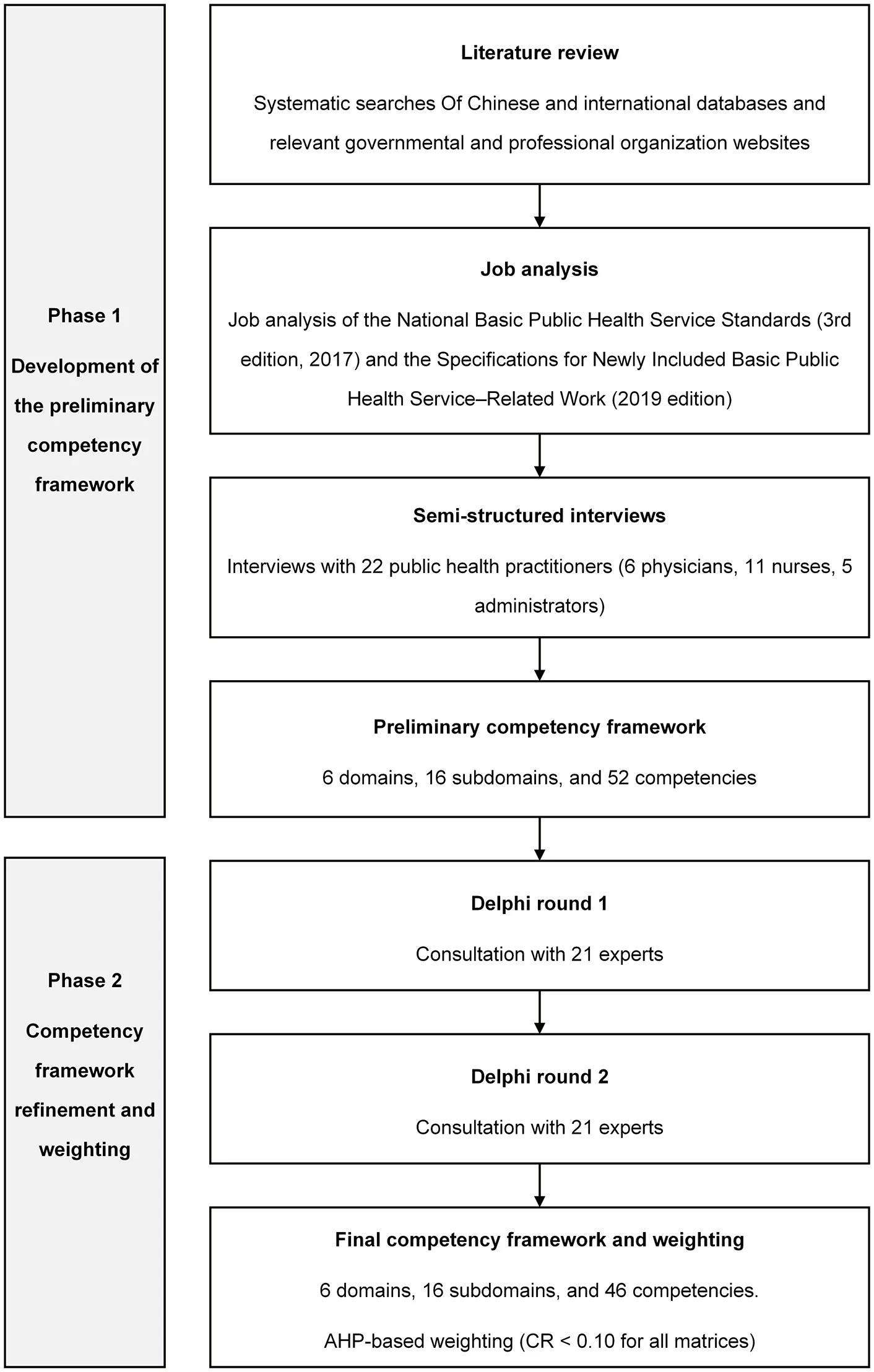
Flowchart of the modified Delphi process.

### Theoretical foundation and conceptual assumptions

This study was underpinned by three complementary theoretical frameworks. The Minnesota Public Health Intervention Wheel [17] provides a population-based public health nursing practice model, encompassing three levels of intervention (individual, community, and system) and 17 evidence-informed public health interventions. The Chinese functional framework for BPHS [10] defines the local public health service system, with the overarching goal of promoting equitable access to BPHS, and specifies eight core service functions, such as health literacy improvement and health information management. The International Council of Nurses (ICN) Competency Framework for Generalist Nurses [20] outlines three core domains: professional, ethical, and legal practice; care provision and management; and professional, personal, and quality development. These domains provide a generic foundation for specialized nursing competencies. Together, these frameworks guided the development of the preliminary competency framework by integrating public health nursing practice, the local public health service context, and generalist nursing competencies.

Existing research suggests substantial overlap between the conceptual definitions and practice scopes of “community nursing” and “public health nursing”. Based on our previous international comparative study [21], the relationship between the two concepts can be broadly categorized into two models: under a generalist model, the two concepts are largely aligned and used interchangeably [22,23]; under a specialist model, public health nursing is regarded as a specialized branch of community nursing [24]. Given China’s developmental trajectory and its “six-in-one” community health service framework—an integrated service model encompassing prevention, health maintenance, medical care, rehabilitation, health education, and technical guidance for family planning—public health nursing was conceptualized as a specialized practice domain within the broader community nursing system. Within this domain, PHNs primarily provide public health-related services, including prevention, health maintenance, health education, and family planning. Drawing on the ICN Framework of Competencies for the Nurse Specialist [25], competency in this study is defined as the systematic integration of knowledge, skills, judgment, and personal attributes (including attitudes, values, and beliefs) required for professionals to fulfill their role responsibilities and achieve desired practice outcomes.

### Development of the preliminary competency framework

#### Literature review.

Using Medical Subject Headings (MeSH) and free-text terms (e.g., “public health nurse,” “community health nurse,” “public health nursing,” “community health nursing,” “professional competence,” “clinical competence,” “framework,” “model,” “assessment,” and “instrument”), we conducted a systematic search of Chinese and international databases, including CNKI, Wanfang Data, VIP, PubMed, Embase, and Web of Science, from inception to April 2022. Detailed search strategies for each database were provided in S2 File. We also performed supplementary searches of the websites of public health governmental agencies and professional organizations worldwide (e.g., the American Association of Public Health Nurses, the Community Health Nurses of Canada, and the National Nursing Academy of Singapore) to identify policy documents, standards, and related materials. In addition, the reference lists of the included publications were screened to identify additional relevant literature not retrieved through the database searches. Publications were included if they (1) were publicly available and relevant to public health nursing competencies, or (2) were reports or documents on competencies issued by official or academic organizations. Exclusion criteria were: (1) non-specific or unclear descriptions of competency items; (2) duplicate publications; (3) non-research items (e.g., editorials and conference abstracts); and (4) publications without accessible full text. Two reviewers independently screened the titles and abstracts of the retrieved records, followed by full-text assessment for potentially eligible publications. Any disagreements were resolved through discussion until a consensus was reached.

#### Job analysis.

Public health nursing services in China are currently delivered through two sets of policy-defined service items: (1) the 12 BPHS items specified in the National Basic Public Health Service Standards (Third Edition, 2017) [26], which define the core role responsibilities and position requirements for PHNs; and (2) the 19 public health service–related tasks set out in the Specifications for Newly Included Basic Public Health Service–Related Work (2019 edition) [27]. The latter are not restricted to primary health care institutions and further enrich the core content of BPHS. A document-based job analysis was conducted using these two policy documents to identify PHNs’ roles, responsibilities, and service tasks. These were interpreted as the knowledge, skills, and abilities required for public health nursing practice and expressed as competency items. The analysis was led by an associate professor with expertise in community nursing research and independently cross-checked by trained graduate nursing students to enhance the consistency of the analysis.

#### Semi-structured interviews.

A qualitative descriptive approach [28] was adopted to explore practitioners’ perspectives on public health nursing competency requirements. The semi-structured interviews were conducted by a graduate nursing student with a background in community nursing research and training in qualitative research methods. No prior relationship was established between the interviewer and participants. Before the interviews, participants were informed of the study's purpose, procedures, and the voluntary nature of participation.

We used maximum-variation purposive sampling [29] to recruit public health practitioners for in-depth interviews from seven primary health care settings, comprising urban, urban-rural fringe, and township facilities, across two cities in Sichuan Province, to capture diverse perspectives across different practice settings. Inclusion criteria were: (1) holding a valid physician or nurse practice license; (2) at least three years of experience in public health–related work; and (3) willingness to participate and ability to communicate effectively. Practitioners who were on leave or attending off-site training during the study period were excluded. Sampling continued until data saturation was achieved, defined as the point at which no new themes emerged from the interviews.

The interview guide was developed for three key practitioner groups—community physicians, nurses, and nursing managers—and covered four domains: (1) service provision and job responsibilities; (2) perceptions of public health nursing practice, including current competencies and practice challenges; (3) competencies considered necessary for effective public health nursing practice; and (4) approaches to strengthening public health nursing capabilities and future team development. The detailed interview guide was provided in S3 File. The guide was pilot tested with two public health practitioners (one physician and one nurse), and minor revisions were made to improve the clarity and comprehensibility of the questions. All interviews were conducted face-to-face in a private office at the participating institutions with only the interviewer and participant present. They were audio-recorded and transcribed verbatim within 48 hours, with relevant non-verbal cues documented in the transcripts.

The interview data were analyzed using thematic analysis in NVivo 11. Themes were identified through an iterative process of coding, categorization, and refinement. Two researchers with qualitative research training independently coded the transcripts, and discrepancies were resolved through discussion until consensus was reached. The completed COREQ checklist [30] was provided as S4 File to support transparent reporting of the qualitative component.

### Delphi expert consultation

#### Expert panel selection.

Experts were recruited from the four major economic regions of China—eastern, central, western, and northeastern—according to the National Bureau of Statistics regional classification [31]. Inclusion criteria were: (1) at least 10 years of relevant professional experience in community nursing, general practice, or public health; (2) holding an intermediate or higher professional title; and (3) willingness to participate.

#### Development of the consultation questionnaire.

Based on the preliminary competency framework, a Delphi questionnaire was developed with four sections: (1) an introduction summarizing the study background, objectives, significance, and prior work; (2) conceptual definitions of key terms, including “public health nursing” and “competency”; (3) a rating section in which experts scored each item on a 5-point Likert scale and suggested additions or revisions; and (4) an expert profile section capturing demographic characteristics, the basis for judgments, and self-assessed topic familiarity.

#### Delphi procedures.

We conducted a two-round Delphi survey from December 2022 to February 2023. Questionnaires were distributed and collected online via the WeChat platform, and experts independently provided their judgments within a one-week response window for each round. Following the first round, we statistically analyzed the importance ratings and thematically synthesized experts’ qualitative comments. Informed by these results and team discussion, the competency items were revised, removed, merged, or added to form the second-round questionnaire. Consensus was assessed using both quantitative and qualitative criteria. Consistent with previous Delphi studies [32], the process concluded when the mean importance rating for each item was ≥ 4.0, the coefficient of variation (CV) was ≤ 0.25, and expert comments were largely limited to minor wording refinements.

#### Statistical analysis and item weighting.

Data were analyzed using SPSS Statistics 26.0. Demographic characteristics were described using means and standard deviations (mean ± SD), frequencies and percentages. Expert engagement, expert authority coefficient, and coordination of expert opinions were evaluated to characterize the Delphi consultation process. Expert engagement was assessed using questionnaire response rates and the proportion of experts providing qualitative feedback. The expert authority coefficient (Cr) was calculated as Cr = (Ca + Cs)/2, where Ca represents the judgment basis coefficient and Cs represents the familiarity coefficient. Ca was derived from four judgment bases—practical or research experience (0.40/0.30/0.20), theoretical knowledge and analysis (0.30/0.20/0.10), relevant literature (0.20/0.10/0.10), and intuition (0.10/0.10/0.10)—with values corresponding to high, moderate, and low influence on expert judgment, respectively. Cs was derived from self-assessed familiarity, with values of 1.00, 0.80, 0.60, 0.40, and 0.20 assigned from very familiar to unfamiliar, respectively. Coordination of expert opinions was assessed using Kendall’s coefficient of concordance (W). A two-sided *P* value ≤ 0.05 was considered statistically significant.

The Analytic Hierarchy Process (AHP) was applied as a structured multi-criteria decision-making approach to determine the relative weights of the items at each level based on expert judgments. The importance ratings provided by Delphi experts were aggregated by calculating the mean scores of each item. Pairwise comparison matrices were constructed by converting the differences between mean importance scores into Saaty’s 1–9 scale [33]. The weights were calculated using SPSSAU online analysis software. The consistency of the judgment matrices was assessed, with a consistency ratio (CR) < 0.10 indicating acceptable consistency.

### Ethical considerations

The study was approved by the Biomedical Ethics Review Committee of West China Hospital, Sichuan University (Approval No. 2022−1058), and written informed consent was obtained from all participants.

## Results

### Preliminary competency framework

In the literature review, 9,547 records related to public health nursing were identified. After removing 3,628 duplicate records, 5,833 records were excluded following title and abstract screening, and a further 46 records were excluded after full-text review. A total of 40 publications (17 in Chinese and 23 in English) were included in the development of the competency framework. The PRISMA-style flow diagram of the literature search and study selection process was presented in S1 Fig. The competency elements identified from the included studies were summarized in S1 Table.

The job analysis of national BPHS programs generated the following candidate competency elements: health information management, health education, health promotion, preventive immunization, population-focused health management, health management for NCDs and mental disorders, health management for patients with tuberculosis, traditional Chinese medicine (TCM)–based health care, public health emergency response, food and environmental safety supervision, teamwork, and communication and coordination.

In the semi-structured interviews, 22 public health practitioners were interviewed, including six physicians, 11 nurses, and five administrators. All participants completed the interviews without withdrawal. Participants were predominantly female (86.36%), with a mean age of 37.64 ± 7.18 years and 13.73 ± 7.07 years of primary health care experience. The mean interview duration was 34 minutes. Participants’ professional roles covered the full range of BPHS programs. Iterative thematic analysis and synthesis yielded 12 preliminary competency elements: health education; health communication; health information analysis and interpretation; population-focused health management (older adults, pregnant women and children); condition-specific health management (NCDs, infectious diseases and severe mental disorders); TCM health management; emergency preparedness and response for public health emergencies; teamwork; interpersonal communication; professional responsibility; quality management; and research.

These data were collated using standardized extraction forms. Guided by theoretical frameworks and informed by integrated data, we conducted a directed content analysis to identify and classify competency elements. This process yielded a preliminary three-tier competency framework for public health nursing, comprising six domains, 16 subdomains, and 52 competencies, which served as the basis for the first-round Delphi questionnaire (S5 File).

### Expert panel characteristics and Delphi process evaluation

#### Expert panel characteristics.

We invited 22 experts to participate in a two-round Delphi consultation. One expert did not return the first-round questionnaire, and the remaining 21 completed both rounds. The panel members were drawn from ten provinces and province-level municipalities across China, including Sichuan, Chongqing, Beijing, Jiangsu, Hunan, and Jilin. The majority of experts were female (80.95%), with a mean age of 49.05 ± 7.62 years and a mean professional experience of 26.48 ± 9.59 years. Overall, 76.19% held a master’s or doctoral degree, and 95.24% held senior professional titles. Most experts were nurses (66.67%), while fields of expertise across the panel included community nursing, public health, primary care medicine, and nursing education and related specialties (Table 1).

**Table 1:** Characteristics of the Delphi expert panel (n = 21).

Variables	Classification	Number	Percentage (%)
**Gender**	Male	4	19.05
	Female	17	80.95
**Age**	≤40	2	9.52
	41-50	12	57.14
	>50	7	33.33
**Years of professional experience**	≤10	2	9.52
	11-20	4	19.05
	21-30	8	38.10
	>30	7	33.33
**Education background**	Bachelor’s degree	5	23.81
	Master’s degree	7	33.33
	Doctoral degree	9	42.86
**Professional title**	Senior	13	61.90
	Associate senior	7	33.33
	Intermediate	1	4.76
**Profession**	Nurses	14	66.67
	Physicians	3	14.29
	Public health physicians	4	19.05
**Field of expertise**	Community nursing	10	47.62
	Public health	5	23.81
	Primary care medicine	2	9.52
	Nursing education and related specialties	4	19.05
**Region**	Eastern China	5	23.81
	Central China	2	9.52
	Western China	11	52.38
	Northeastern China	3	14.29

#### Expert engagement.

In the first round, 21 of 22 experts responded (response rate, 95.45%), and 17 (80.95%) provided substantive revision suggestions. In the second round, all 21 experts responded (response rate, 100%), and 9 (42.86%) offered additional constructive comments.

#### Expert authority coefficient.

In this study, Ca and Cs were 0.92 and 0.87, respectively, yielding an overall Cr of 0.90.

#### Coordination of expert opinions.

Kendall’s W values for the two rounds were 0.156 and 0.171, respectively, and both were statistically significant (*P* < 0.001; Table 2).

**Table 2:** Degree of expert consensus.

Round	Kendall’s W	*χ* ^2^	*P* value
1	0.156	166.668	<0.001
2	0.171	172.025	<0.001

### Refinement and weighting of competency framework

#### Framework refinement and finalization.

In the first Delphi round, the mean importance ratings for all items ranged from 4.00 to 5.00, with CVs between 0.00 and 0.24; thus, all items met the predefined quantitative criteria. Nevertheless, experts provided substantive qualitative comments on the content and structure of the framework. Each suggestion was reviewed by the research team, and modifications were made following team discussion, considering conceptual overlap, clarity and precision of wording, and consistency with the defined scope of public health nursing. Relevant policy documents, literature, and findings from the preliminary fieldwork were also considered when applicable. Based on this process, six competencies were removed; one domain, four subdomains, and two competencies were revised; four new competencies were added; one pair of subdomains and two pairs of competencies were merged; one subdomain was split; and the wording of several definitions was refined accordingly. The revised framework formed the basis of the second-round Delphi questionnaire (S6 File). In the second round, the mean importance ratings increased to a range of 4.38 to 5.00, while CVs narrowed to between 0.00 and 0.18. Fewer experts provided qualitative feedback, which mainly focused on further refinement of the framework. Following review and research team discussion, two competencies were removed because of conceptual overlap, and minor textual adjustments were made to several definitions. The pre-specified stopping criteria were met; therefore, no further Delphi rounds were conducted. The final competency framework for public health nursing thus comprised six domains, 16 subdomains, and 46 competencies (Table 3). Operational definitions of all competencies were provided in S2 Table. In addition, to facilitate the educational interpretation of the competency framework, the competencies were mapped to Bloom's revised taxonomy [34] as an interpretative layer (S3 Table).

**Table 3:** Final competency framework for public health nursing: importance ratings, coefficients of variation, and weights.

Competency framework	Importance rating, mean ± SD	CV	Weight	Global weight
**1. Community health surveillance and assessment**	4.90 ± 0.30	0.06	0.196	0.196
1.1 Community health surveillance	4.76 ± 0.54	0.11	0.333	0.065
1.1.1 Knowledge of health surveillance	4.62 ± 0.67	0.14	0.198	0.013
1.1.2 Ability to collect and manage health surveillance data	4.81 ± 0.51	0.11	0.312	0.020
1.1.3 Ability to report and communicate health surveillance information	4.86 ± 0.48	0.10	0.491	0.032
1.2 Community health assessment	4.95 ± 0.22	0.04	0.667	0.130
1.2.1 Ability to conduct comprehensive community nursing assessments	4.90 ± 0.30	0.06	0.333	0.043
1.2.2 Ability to develop community nursing diagnoses	4.95 ± 0.22	0.04	0.667	0.087
**2. Health education and health promotion**	4.95 ± 0.22	0.04	0.280	0.280
2.1 Health education	5.00	0.00	0.667	0.187
2.1.1 Ability to design health education programs	5.00	0.00	0.286	0.053
2.1.2 Ability to develop health education materials	5.00	0.00	0.286	0.053
2.1.3 Ability to implement health education programs	5.00	0.00	0.286	0.053
2.1.4 Ability to evaluate health education programs	4.76 ± 0.54	0.11	0.143	0.027
2.2 Health promotion	4.86 ± 0.36	0.07	0.333	0.093
2.2.1 Ability to engage in health advocacy	4.81 ± 0.40	0.08	0.667	0.062
2.2.2 Ability to conduct community mobilization	4.76 ± 0.44	0.09	0.333	0.031
**3. Health management**	4.90 ± 0.30	0.06	0.196	0.196
3.1 Health information technology	4.90 ± 0.30	0.06	0.221	0.043
3.1.1 Ability to use health information systems	4.90 ± 0.30	0.06	0.667	0.029
3.1.2 Ability to deliver internet-enabled health services	4.86 ± 0.36	0.07	0.333	0.014
3.2 Population-focused health management	4.90 ± 0.30	0.06	0.221	0.043
3.2.1 Ability to manage the health of children aged 0–6 years	4.86 ± 0.48	0.10	0.250	0.011
3.2.2 Ability to manage women’s health	4.86 ± 0.48	0.10	0.250	0.011
3.2.3 Ability to manage older adults’ health	4.90 ± 0.30	0.06	0.500	0.022
3.3 Condition-specific health management	4.95 ± 0.22	0.04	0.339	0.066
3.3.1 Ability to manage the health of people with NCDs	4.90 ± 0.30	0.06	0.400	0.027
3.3.2 Ability to manage the health of people with infectious diseases	4.90 ± 0.30	0.06	0.400	0.027
3.3.3 Ability to manage the health of people with mental disorders	4.86 ± 0.36	0.07	0.200	0.013
3.4 Setting-specific health management	4.71 ± 0.56	0.12	0.137	0.027
3.4.1 Ability to manage health in school settings	4.62 ± 0.74	0.16	0.667	0.018
3.4.2 Ability to manage health in workplace settings	4.38 ± 0.80	0.18	0.333	0.009
3.5 TCM health management	4.48 ± 0.68	0.15	0.081	0.016
3.5.1 Knowledge of TCM health management	4.43 ± 0.68	0.15	0.333	0.005
3.5.2 Ability to provide TCM‑based health guidance	4.52 ± 0.68	0.15	0.667	0.011
**4. Community public health emergency management**	4.86 ± 0.48	0.10	0.110	0.110
4.1 Risk management	4.86 ± 0.48	0.10	0.312	0.034
4.1.1 Ability to conduct community risk assessments	4.81 ± 0.51	0.11	0.297	0.010
4.1.2 Ability to develop emergency response plans	4.57 ± 0.81	0.18	0.164	0.006
4.1.3 Ability to provide community education on emergency management	4.90 ± 0.44	0.09	0.539	0.018
4.2 Emergency response	4.90 ± 0.44	0.09	0.491	0.054
4.2.1 Ability to report emergency information	4.81 ± 0.68	0.14	0.133	0.007
4.2.2 Ability to implement public health emergency response measures and deliver emergency medical care	4.90 ± 0.44	0.09	0.289	0.016
4.2.3 Ability to conduct epidemiological investigations	4.71 ± 0.56	0.12	0.089	0.005
4.2.4 Ability to collect clinical specimens	4.86 ± 0.48	0.10	0.189	0.010
4.2.5 Ability to support containment and environmental decontamination measures in outbreak-affected areas	4.86 ± 0.48	0.10	0.189	0.010
4.2.6 Ability to provide psychological first aid	4.76 ± 0.54	0.11	0.109	0.006
4.3 Recovery-phase management	4.71 ± 0.56	0.12	0.198	0.022
4.3.1 Ability to conduct post-event evaluation	4.76 ± 0.70	0.15	0.500	0.011
4.3.2 Ability to manage recovery-phase health issues	4.76 ± 0.70	0.15	0.500	0.011
**5. Partnership development and maintenance**	4.86 ± 0.48	0.10	0.110	0.110
5.1 Teamwork	4.86 ± 0.48	0.10	0.500	0.055
5.1.1 Ability to establish and sustain collaboration with external community partners	4.62 ± 0.67	0.14	0.333	0.018
5.1.2 Ability to collaborate within the community health team	4.86 ± 0.48	0.10	0.667	0.037
5.2 Communication and coordination	4.86 ± 0.48	0.10	0.500	0.055
5.2.1 Ability to conduct effective communication	4.86 ± 0.48	0.10	0.667	0.037
5.2.2 Ability to align stakeholders	4.76 ± 0.62	0.13	0.333	0.018
**6. Professionalism and professional development**	4.86 ± 0.48	0.10	0.110	0.110
6.1 Professionalism	4.81 ± 0.51	0.11	0.667	0.073
6.1.1 Professional responsibility	4.86 ± 0.48	0.10	0.279	0.020
6.1.2 Legal literacy	4.81 ± 0.51	0.11	0.165	0.012
6.1.3 Ethical literacy	4.90 ± 0.44	0.09	0.392	0.029
6.1.4 Humanistic literacy	4.81 ± 0.60	0.13	0.165	0.012
6.2 Professional development	4.76 ± 0.54	0.11	0.333	0.037
6.2.1 Ability to engage in self-directed learning	4.71 ± 0.56	0.12	0.244	0.009
6.2.2 Ability to teach and mentor others	4.71 ± 0.64	0.14	0.244	0.009
6.2.3 Ability to conduct continuous quality improvement	4.81 ± 0.51	0.11	0.413	0.015
6.2.4 Ability to contribute to public health research projects	4.38 ± 0.67	0.15	0.099	0.004

Abbreviations: SD, standard deviation; CV, coefficient of variation; NCDs, non-communicable diseases; TCM, traditional Chinese medicine.

#### Framework weighting and consistency.

Among the six domains, health education and health promotion was assigned the highest weight (0.280), followed by health management and community health surveillance and assessment (both 0.196), and community public health emergency management, partnership development and maintenance, and professionalism and professional development (all 0.110). All judgment matrices exhibited acceptable consistency (CR < 0.10; Table 3).

## Discussion

### Methodological rigor of the competency framework development

This study used a modified Delphi approach to systematically develop a competency framework for public health nursing in China.

At the conceptual level, the framework integrated three complementary perspectives: the Minnesota Public Health Intervention Wheel, the “1+8” functional structure of China’s BPHS, and the ICN competency standards for generalist nurses. This integration aligned the framework with international competency-based development while ensuring responsiveness to China’s service delivery realities.

At the methodological level, the identification and generation of competency items were informed by a multi-source triangulation strategy. The literature review synthesized findings from domestic and international studies on public health nursing competencies. The job analysis explicitly mapped national BPHS standards to competency requirements, thereby enabling a clear translation of policy-mandated tasks into competency statements. Furthermore, semi-structured interviews with 22 frontline practitioners (physicians, nurses, and managers), recruited via maximum variation sampling from diverse primary health care settings, helped to broaden content coverage and strengthen contextual relevance.

During the Delphi process, we assembled a panel of 21 experts in community nursing, public health, primary care medicine, and nursing education and related specialties from four major economic regions in China. The panel was highly qualified, with 76.19% holding a master’s degree or higher and 95.24% possessing senior professional titles. The methodological quality of the Delphi process was further supported by sustained engagement across rounds (response rates: 95.45% in Round 1 and 100% in Round 2) and a high expert authority coefficient (Cr = 0.90). Although Kendall’s W values were statistically significant, their relatively low magnitudes (0.156 and 0.171) suggested limited agreement on the prioritization of competencies. This limited overall concordance may partly reflect a potential ceiling effect from consistently high importance ratings, which may have reduced the ability of the rating scale to discriminate among items. In contrast, the decreasing CV values (from ≤0.24 to ≤0.18) indicated improved convergence in experts’ ratings, and item-level consensus on the inclusion of individual competencies was achieved based on the predefined criteria.

Overall, the integration of theoretical foundations, multi-source evidence, and iterative expert consultation based on predefined criteria supported the methodological rigor of the framework development.

### Content and weighting analysis of the competency framework

#### Community health surveillance and assessment.

Community health monitoring and assessment was grounded in the Minnesota Public Health Intervention Wheel [17] and operationalized within China’s BPHS functional framework [10]. It was further contextualized by insights from public health nursing frameworks in the United States [14], Canada [15], and Japan [16]. This domain involves the systematic collection, integration, and analysis of information on the community environment and population health to produce actionable assessments. As a foundational area of public health nursing practice, it underpins priority setting, intervention planning, and evaluation, and is classified as a community-level professional competency in frameworks used in Minnesota [17] and Singapore [35]. In this study, this dimension received a weighting coefficient of 0.196, ranking second among all domains, highlighting its pivotal role within the overall framework. This finding suggests that it should be prioritized in training initiatives and resource allocation to efficiently promote the community health.

#### Health education and health promotion.

Health education and health promotion are widely recognized as among the most fundamental, cost-effective, and impactful strategies for disseminating health knowledge, fostering healthy lifestyles, and improving population health outcomes. Health education supports individuals and groups in making informed health decisions through structured education and communication. Health promotion extends beyond individual-level change to advance healthier ways of living through broader policy action and multisectoral governance. Health education underpins health promotion and is integral to its processes [36]. In our study, health education was informed by the Green model [37] and conceptualized as a program cycle of planning and design, implementation, and evaluation. Health promotion was defined in a narrower sense [38] as the ability of health-system professionals to advocate for multisectoral support and mobilize population participation to promote health. Specifically, advocacy aimed to shape an enabling policy environment, whereas mobilization sought to activate community engagement and motivation [39]. This dimension was assigned the highest weighting coefficient (0.280) among the six domains. Within China's National BPHS, health education is both a designated service and an integral component of chronic disease management and other population-focused health services. This cross-cutting role and its close alignment with routine frontline responsibilities may partly explain its relatively high weight. This prioritization is consistent with the established role of PHNs as key agents in health education and promotion [40,41]. Accordingly, strengthening this competency may contribute to improving the quality, impact, and sustainability of community-based public health services.

#### Health management.

Community health management is an integrated, ongoing process of monitoring, assessing, intervening in, and following up on the health status and risk factors of community residents. As a core component of China’s BPHS program, it operationalizes a shift toward earlier identification, proactive intervention, and sustained management in disease prevention and control. Within this domain, five subdomains are specified in accordance with China’s national context and service priorities: (1) health information technology; (2) population-focused health management; (3) condition-specific health management; (4) setting-specific health management; and (5) TCM health management. Together, these components provide broad coverage of essential community health management activities, supporting the framework’s integrity, relevance, and practical applicability in routine primary care and public health practice.

Within the health management domain, TCM health management received a relatively low subdomain weight (0.081) and global weight (0.016). Nevertheless, its mean importance rating was 4.48, with mean ratings of 4.43 and 4.52 for knowledge of TCM health management and the ability to provide TCM-based health guidance, respectively, indicating that these competencies were still considered important by the expert panel. Although TCM health management is included in the National BPHS, the relevant service standards specify professional qualification and training requirements for specific TCM-related services [26]. In this context, PHNs may participate in information collection and constitution identification, support service coordination, and reinforce guidance provided by appropriately qualified practitioners. This distinction in professional roles may partly explain the relatively low weight assigned to TCM health management.

#### Community public health emergency management.

Community public health emergency management is essential for safeguarding residents’ lives, health, and safety [8,42]. This dimension was derived from China’s BPHS functional framework [10] and structured according to the disaster management cycle theory [43]. It comprises three subdomains: risk management, emergency response, and recovery-phase management. Among these, emergency response competency received the highest global weight (0.054), consistent with findings from Lin et al. [44] on community nurses’ disaster response competencies. This finding highlights the centrality of emergency response in community public health emergency management. However, effective response also depends on robust preparedness and systematic post-event recovery. Capacity-building efforts should therefore adopt an integrated, cyclical approach in which preparedness enables timely response, and recovery informs subsequent preparedness.

#### Partnership development and maintenance.

In the context of population aging and the rising burden of chronic disease, the increasing complexity of public health service demands often exceeds the capacity of any single provider, making cross-sector collaboration imperative. In response, our study identified partnership development and maintenance as a domain of the competency framework. It reflects the application of a collaborative approach to integrating and coordinating disparate community resources into a coherent, well-functioning system, thereby enabling the effective delivery of public health services. This domain consists of two interrelated subdomains: teamwork, and communication and coordination. Both are consistently recognized in prior studies as essential attributes of PHNs [45,46] and are critical to operationalizing community collaboration by facilitating stakeholder engagement, clarifying roles, and establishing coordinated workflows.

#### Professionalism and professional development.

Professionalism and professional development were grounded in the ICN framework for generalist nurses [20]. Together, they reflect essential attributes for public health nursing practice and the capacity for ongoing professional growth. In this study, professionalism received a higher global weight (0.073) than professional development (0.037). This weighting pattern contrasts with findings from Zhai et al. [47]. A possible explanation relates to the developmental stage of public health nursing in China: professionalism may currently be emphasized as a baseline requirement, whereas professional development may be perceived as a value-added one. Accordingly, education and in-service training should prioritize embedding role-essential competencies into core curricula, while progressively integrating structured professional development pathways as the workforce and service system evolves.

### International comparison of the competency framework

Internationally, public health nursing competency frameworks are typically characterized by systematic organization, hierarchical structures, and professional specificity. The United States employs a public health nursing competency framework differentiated across three practice levels (entry, supervisory, management), with 79 statements across eight domains, including assessment and analysis, policy and program planning, communication, cultural competence, community practice, public health sciences, financial management, and systems leadership [14]. Canada similarly outlines 66 statements across eight domains, covering public health and nursing sciences; assessment and analysis; policy and program planning, implementation, and evaluation; partnerships, collaboration, and advocacy; diversity and inclusiveness; communication; leadership; and professional responsibility and accountability [15]. Japan identifies a set of core competencies spanning professional accountability, application of information technology, community health assessment and analysis, health promotion and prevention, policy and program implementation, healthy community management, collaboration, and communication [16]. Taken together, these frameworks emphasize broad professional competencies in assessment and analysis, policy and program planning, communication and collaboration, and leadership across diverse public health nursing roles and practice settings.

Compared with international models, the competency framework developed in this study retains these shared professional core elements while demonstrating distinct local adaptation. It incorporates competencies closely aligned with frontline service delivery under China’s National BPHS, consistent with the current responsibilities of PHNs in implementing standardized public health services within primary health care settings. This emphasis on frontline practice may help explain why policy development, financial management, and systems leadership were not included as separate domains. The framework nevertheless incorporates elements of program management, policy engagement, and collaboration through competencies in health education planning and evaluation, health advocacy, community mobilization, and communication and coordination. Further refinement of the framework should clarify competency requirements for policy development, financial management, and systems leadership across different professional roles and career stages. Furthermore, the inclusion of a dedicated domain on community public health emergency management reflects the role of PHNs in frontline response and community resilience during public health crises. This dimension aligns with the national policy mandate to build an integrated public health system for both routine and emergency contexts, demonstrating the framework’s responsiveness to policy priorities and operational realities.

### Implications for PHN workforce development

This study established a competency framework for public health nursing to address the current lack of a systematic, context-specific framework for PHNs in China. The defined competencies provide a structured reference for aligning education and in-service training with the requirements of primary public health practice. As the PHN workforce develops, future work should establish tiered competency levels across stages of professional development, potentially informed by models such as Kuehnert’s public health nursing practice model [48]. This could provide a basis for standardized training and professional development pathways and inform related workforce policies in China.

### Limitations

This study has several limitations. First, the preliminary qualitative interviews were conducted in seven primary health care settings in two cities in Sichuan Province. Despite the use of maximum-variation sampling, the geographically concentrated sample may have limited the diversity of perspectives captured and the transferability of the qualitative findings. Potential social desirability and interviewer influence during the interviews also cannot be entirely excluded. Second, although experts from four major economic regions of China were included, more than half were from Western China, and community nursing remained the largest disciplinary group within the multidisciplinary Delphi panel. These factors may affect the representativeness and comprehensiveness of the framework. Finally, the framework was developed through expert consensus and requires further empirical validation in practice, while the competencies remain to be translated into measurable learning outcomes. Future research should validate and refine the framework by involving broader regional and multidisciplinary samples, conducting pilot implementation and psychometric evaluation in practice settings, and developing measurable learning outcomes, thereby supporting its application in nursing education, practice, and policy development.

## Conclusion

This study used a modified Delphi approach to develop a competency framework for PHNs in China, informed by the current practice context and evolving service needs. The final framework comprises six domains, 16 subdomains, and 46 competencies. The framework provides a structured reference for PHN education, in-service training, and professional development and may contribute to strengthening the PHN workforce in China.

## Supporting information

S1 File Guidance on Conducting and Reporting Delphi Studies (CREDES) Checklist. (DOCX)

S2 File Detailed literature search strategies. (DOCX)

S3 File Semi-structured Interview Guide [English Translation]. (DOCX)

S4 File Consolidated Criteria for Reporting Qualitative Research (COREQ) Checklist. (DOCX)

S1 Fig PRISMA-style flow diagram of the literature search and study selection process. (TIF)

S1 Table Summary of competency elements identified from the literature review. (DOCX)

S5 File Expert Consultation Questionnaire for the Development of a Public Health Nursing Competency Framework (Round 1) [English Translation]. (DOCX)

S6 File Expert Consultation Questionnaire for the Development of a Public Health Nursing Competency Framework (Round 2) [English Translation]. (DOCX)

S2 Table Public Health Nursing Competency Framework with Operational Definitions of Competencies. (DOCX)

S3 Table Mapping of competencies in the public health nursing competency framework to Bloom’s revised taxonomy. (DOCX)
