# Supplementary material for: Development of a competency framework for public health nurses in China: A modified Delphi study
Source: PLoS One. 2026 Oct 30;21(10):e0361940. doi: 10.1371/journal.pone.0361940 (PMC13722293; doi:10.1371/journal.pone.0361940)
Supplement: Supplementary file 1 [file pone.0361940.s011.pdf]

Dear Authors,

Thank you for the opportunity to review this manuscript.

This manuscript reports the development of a competency framework for public health nurses (PHNs) in China using a modified Delphi approach combined with Analytic Hierarchy Process (AHP). A preliminary three-tier framework was constructed from a literature review, a job document analysis and semi-structured interviews with 22 frontline health practitioners. This preliminary framework was then refined through two rounds of Delphi process (with a panel of 21 experts), and AHP was applied to assign relative weights to the agreed items. The final framework comprises six primary items, 16 secondary items, and 46 tertiary items.

The study addresses a timely and relevant gap. The need for a systematic, context-specific competency framework for PHNs in China is well justified, particularly in light of the country's evolving public health landscape, the rising burden of chronic disease, and the lessons drawn from successive infectious disease outbreaks. The professionalization of the PHN workforce is, moreover, a recognized national priority (e.g., Healthy China 2030 and the 14th Five-Year Plan), yet, as the authors note, no systematic, context-specific PHN competency framework currently exists. Aligning workforce development with both national service standards and internationally recognized competency models therefore represent a meaningful contribution, with clear implications for nursing education, training, and policy.

From a methodological standpoint, the study has several strengths. First, the use of a multi-source triangulation strategy (combining a literature review, a job document analysis, and interviews with health practitioners) to construct the preliminary framework is a commendable and well-justified design choice that grounds the items in both policy mandates and frontline realities. Second, the explicit anchoring of the framework in China's Basic Public Health Service (BPHS) standards strengthens its local relevance and practical applicability while retaining the globally recognized core domains of public health nursing. Third, the reported adherence to the CREDES reporting checklist further supports the rigour of the Delphi process.

Despite these strengths, the manuscript would benefit from thorough revision to improve its methodological transparency and clarity. Having read the manuscript twice, I outline below a number of comments and suggestions for the authors' consideration.

Major issues/Major concerns

1. **Introduction. Rationale asserted rather than substantiated.** The statement that *"(...) because of differences in healthcare systems, sociocultural contexts, and stages of development, these frameworks cannot be directly applied in China"* (lines 103–105) is presented as a given. As this assertion is central to the rationale for the study, it would benefit from substantiation rather than being stated as self-evident. The argument would be considerably strengthened were the authors to elaborate on this claim by identifying the specific differences, and similarities, between the international frameworks (e.g., those of the United States, Canada, and Japan) and the Chinese context - for instance, in the organization of primary care, the role boundaries between public health and community nursing, and the sociocultural

expectations of the nursing role - and by explaining how each of these factors would bear upon the design and implementation of a competency framework for PHNs in China. Such an elaboration would render the case for a China-specific framework explicit rather than assumed.

2. **Cross-cutting issue. Reconsider the use of the term "indicators."** The framework is described as comprising *"six primary domains, 16 secondary indicators, and 46 tertiary indicators."* The term "indicators" may not best convey the constructs being described, which are competencies rather than measurement indicators. Consistent and accurate terminology is important for conceptual clarity and for alignment with the competency frameworks' literature. The authors should reconsider the terminology, for example adopting a hierarchy such as domains → competencies → sub-competencies, and apply it consistently throughout the manuscript.
3. **Methods. Incorrect attribution of the definition of 'competency'**. The definition provided, namely that *"competency is defined as the systematic integration of knowledge, skills, judgment and personal attributes (including attitudes, values and beliefs) that professionals require to fulfil their role responsibilities and achieve desired practice outcomes*" (lines 157–160), is attributed to McClelland, 1973. This attribution appears questionable: McClelland's seminal work introduced the general concept of testing for competence, whereas the wording adopted here corresponds far more closely to the competence definition formulated by the Canadian Nurses Association. Because an accurate conceptual foundation is essential to the validity of the framework, the authors should verify the source and cite the definition correctly.
4. **Methods. Insufficient reporting of the literature review.** The search is described as "systematic," yet no structured search strategy, PRISMA flow diagram, screening protocol, number of reviewers, or procedure for resolving disagreements is reported. Furthermore, only a raw count is provided (9547 records reduced to 40 included), with no breakdown of records excluded at each stage. These omissions prevent readers from appraising the comprehensiveness and reproducibility of the evidence base underpinning the preliminary framework. The authors should report the full search strategy, present a PRISMA-style flow diagram documenting records at each stage, and describe the screening procedure, including the number of reviewers involved and the method for resolving disagreement.
5. **Methods. Specify the type of "job analysis" performed.** The procedure labelled "job analysis" (lines 182–197) derives competency items from two national policy documents (the National Basic Public Health Service Standards, 3rd ed., and the Specifications for Newly Included Basic Public Health Service–Related Work). Because job analysis may be conducted through several distinct methods (e.g., document or standards review, observation, work sampling, or incumbent surveys), the lack of an explicit designation could lead readers to assume that a field-based analysis was undertaken. To aid transparency, the authors should state explicitly that a document-based job analysis was performed, briefly describe how the described job specification roles and tasks were extracted and translated into competency statements, and clarify how this step relates to the subsequent practitioner interviews.
6. **Methods. Lack of an explicit qualitative research design.** The semi-structured interviews are described procedurally, but no underlying theoretical or methodological orientation (e.g., phenomenology, grounded theory, reflexive thematic analysis) is identified. As the chosen orientation shapes the design of the interview guide, the sampling logic, and the mode of analysis, its omission limits the reader's

ability to judge the coherence of the qualitative component. The authors should state the theoretical grounding that informed the conduct and analysis of the interviews.

7. **Methods. COREQ checklist for the interviews.** A completed COREQ (Consolidated Criteria for Reporting Qualitative Research) checklist (Tong et al., 2007) does not appear to be included. Given that the interviews materially shaped the preliminary framework, adherence to an established qualitative reporting standard is important for transparency and appraisal. The authors should complete and provide a COREQ checklist as supporting information.
8. **Methods. Non-nurse perspectives in a nursing competency framework**. The interviewees comprised physicians and administrators in addition to nurses (6 physicians, 11 nurses, 5 administrators), meaning that a framework of nursing competencies is partly grounded in non-nurse perspectives. Without a conceptual justification, it is unclear how the input of non-nurse participants maps onto PHN-specific competencies, which raises a question of construct validity. The authors should justify the inclusion of these groups and explain how their contributions were interpreted and integrated in relation to public health nursing competencies.
9. **Methods — Reporting and trustworthiness of the thematic analysis.** The thematic analysis is reported as having used NVivo 11, but no coding framework, number of coders, measure of inter-coder reliability, or audit trail is described, and trustworthiness criteria (e.g., credibility, confirmability, member checking) are not addressed. The absence of these elements undermines confidence in the rigour of the qualitative analysis. The authors should describe the coding process, the number and roles of coders, the procedures used to establish trustworthiness, and any audit trail maintained.
10. **Methods. Potentially leading interview guide.** The four domains of the interview guide appear partly leading (e.g., "competency requirements… and their specific components"), which may have primed participants toward a pre-existing competency structure rather than eliciting open accounts. This risks circularity, whereby the interviews confirm rather than inform the framework. The authors should reflect on this possibility, and ideally provide the interview guide (see comment 10) so that the degree of openness can be assessed.
11. **Methods. Provide the interview guide.** The interview guide is referred to but not supplied. As the guide is central to appraising the qualitative component and the concern raised in comment 9, its inclusion as supporting information would substantially enhance transparency. The authors should provide the full interview guide.
12. **Methods. Provide the completed CREDES checklist.** The manuscript states that the process followed the CREDES reporting checklist, yet a completed checklist does not appear to be included. Without it, readers cannot verify the extent of adherence to the standard. The authors should provide the completed CREDES checklist as supporting information.
13. **Methods. Rationale for using AHP.** The manuscript applies AHP to derive indicator weights but does not explain why this method was selected in preference to other multi-attribute value measurement approaches. As the choice of weighting method influences the results and their interpretation, a justification is warranted. The authors should briefly explain what AHP is, state why it was chosen over alternative methods, and acknowledge its principal strengths and limitations.

14. **Methods. Transparency of the AHP weighting procedure.** The AHP weighting is underspecified: the Methods state only that "AHP was used to determine the weights." Because the relative weights are a central deliverable of the study, this lack of detail is a significant limitation; it is unclear who performed the pairwise judgments, how many respondents contributed to each matrix, how individual judgments were aggregated, and at what point in the process the comparisons were collected. Since the validity and interpretability of the weights depend entirely on this procedure, its omission constrains appraisal of the results. The authors should describe the AHP elicitation in full, including the respondents and their number, the comparison scale used, the aggregation method, and the manner in which consistency (CR < 0.10) was handled for any matrices that initially failed.
15. **Methods/Results. Low Kendall's coefficient of concordance versus the claim of strong consensus.** The reported Kendall's W values are 0.156 (Round 1) and 0.171 (Round 2). Although both are statistically significant, the significance here is largely a function of the number of items rather than the strength of agreement, and values in this range conventionally indicate only weak overall concordance among experts. This sits in tension with the manuscript's repeated framing of the process as demonstrating "strong expert consensus" and "increasing agreement across rounds," given that the increase from 0.156 to 0.171 is marginal and the absolute level remains low. The authors should (i) explicitly acknowledge and interpret the low W values, (ii) distinguish between item-level consensus (the mean and CV thresholds, which were met) and overall opinion coordination (W, which was weak), and (iii) discuss what the low W implies for the robustness of the framework. Reporting W alongside accepted interpretive benchmarks would substantially improve the credibility of this section.
16. **Methods. Representativeness of the expert panel.** The final panel ($n = 21$) is comparatively small for a framework intended to be nationally applicable and is notably skewed in both geography and discipline: 52.4% of experts were from Western China and only 9.5% from Central China, while 57.1% specialized in community nursing, with public health and general practice represented by only three experts each (14.3%). This imbalance could bias which competencies are prioritized and weighted, thereby limiting the national generalizability of the framework. The authors should explicitly acknowledge this distribution as a limitation.
17. **Results. Output of the literature review**. The Results section do not report which competencies, or how many, emerged from the analysis of the retrieved studies. As the literature review is one of the three pillars of the preliminary framework, this omission leaves a gap. The authors should report the competencies derived from the literature review and their number.
18. **Results. Present the preliminary framework.** The text states that the process "*yielded a preliminary three-tier competency framework for public health nursing, comprising six primary, 16 secondary, and 52 tertiary indicators,"* but this preliminary framework is not presented. Without it, readers cannot trace how the framework evolved through the Delphi rounds. The authors should present the full preliminary three-tier framework (e.g., as a table/figure within the manuscript or as a supplementary material file).
19. **Results. Professional groups of the Delphi participants.** The Delphi results do not detail the professional groups of the expert panel beyond broad fields of expertise. As the credibility of a Delphi study depends heavily on panel composition, detailed reporting is warranted. The authors should describe

the professional groups represented among the participants. For instance, it would be relevant to know how many nurses participated in the Delphi process.

20. **Results. Present the Delphi questionnaires across the several rounds.** The manuscript would benefit from presentation of the web-Delphi questionnaire screens used across the several rounds. Doing so would allow readers to understand how items were presented and rated and would enhance transparency. The authors could include representative questionnaire screens as supporting material.
21. **Results. Calculation of the expert authority coefficient.** The expert authority coefficient (Cr = 0.90) is reported but its method of calculation is not described. As this is a key indicator of the reliability of the consultation, the basis for its computation should be transparent. The authors should specify how the expert authority coefficient, including its components (judgment basis and familiarity), was derived.
22. **Results. Rationale for items changes**. The statement that, *"based on expert feedback and discussion within the research team, six tertiary indicators were removed" (*and that others were revised, merged, or added) does not explain the basis for these decisions. Without explicit decision rules, the modifications may appear post-hoc and reduce procedural transparency. The authors should explain the criteria and reasoning underpinning the removal, addition, merging, and revision of indicators across rounds.
23. **Results. Define the pre-specified stopping criteria**. The sentence "The pre-specified stopping criteria were met; therefore, no further Delphi rounds were conducted" does not state, at that point, what those criteria were. Because the decision to halt the process bears directly on the validity of the claimed consensus, the criteria should be unambiguous. The authors should clearly state the pre-specified stopping criteria and confirm that they were defined a priori.
24. **Discussion. Contributions and implications**. The Discussion would benefit from a clearer articulation of the study's contributions and implications for both research and practice. In particular, it remains underdeveloped how the findings will be used to strengthen PHN workforce development in China. The authors should explain how the framework can inform education, training, assessment, and policy, and discuss the next steps required to operationalize it.
25. **Discussion. Strengthen the Discussion, section including the 'Study Limitations' sub-section.** The Discussion, and particularly the limitations section, is currently underdeveloped; the limitations paragraph addresses only the absence of career-level differentiation. A more comprehensive treatment would strengthen the manuscript and demonstrate balanced appraisal. The authors should expand the Discussion and limitations to address, among others, the occurrence of potential biases concerning the interviewing process (e.g. sampling bias, social desirability bias, interviewer biases) the low Kendall coefficient of concordance, the single-province sampling of the interviews, potential ceiling effects arising from the rating scale or the fact that the framework has not yet been empirically validated.

Minor issues/Minor comments

1. Figure 1 — Typographical error. In the flowchart (Fig 1), "Delphi" is misspelled as "Dephi" in both "Dephi round 1" and "Dephi round 2." Please correct this.
2. Figure 1 - Image resolution. Figure 1 is difficult to read. Given the importance of the figure for conveying the overall study process, please also ensure it is supplied at sufficient resolution for publication.

3. Language and minor grammar issues. A few sentences would benefit from light editing for subject–verb agreement and clarity; for example, "the role of public health nurses (PHNs)…have become increasingly prominent" (lines 81–83) should read "has become." A general language revision is recommended.
