# Supplementary material for: Development of a competency framework for public health nurses in China: A modified Delphi study
Source: PLoS One. 2026 Oct 30;21(10):e0361940. doi: 10.1371/journal.pone.0361940 (PMC13722293; doi:10.1371/journal.pone.0361940)
Supplement: Supplementary file 2 [file pone.0361940.s012.pdf]

Review of Manuscript PONE-D-26-02847

Title: Development of a competency framework for public health nurses in China: A modified Delphi study

Confidential Comments to the Academic Editor of *Plos One*

Dear Dr. Mohd Ismail Ibrahim, Academic Editor of *Plos One,*

This manuscript presents the development of a competency framework for public health nurses (PHNs) in China, combining a modified Delphi approach with the Analytic Hierarchy Process (AHP). A preliminary three-tier framework, derived from a literature review, a job document analysis, and semi-structured interviews with 22 frontline practitioners, was refined through two rounds of expert consultation (21 experts) and weighted using AHP, yielding a final framework of six primary, 16 secondary, and 46 tertiary items. The topic is timely and policy-relevant, addressing a genuine gap, since no systematic, context-specific competency framework for PHNs currently exists in China.

The study has some strengths. The research question aligns with recognized national priorities (e.g., Healthy China 2030, the 14th Five-Year Plan); the multi-source triangulation strategy grounds the framework in both policy mandates and frontline realities; the explicit anchoring in China's Basic Public Health Service standards strengthens local relevance while retaining internationally recognized core domains; and the reported adherence to the CREDES checklist reflects attention to transparency.

Notwithstanding these strengths, the manuscript requires a thorough and substantial revision, as the principal concerns are methodological and extend across all four pillars of the study design, the manuscript requires substantial revision before it can be considered methodologically sound. My main concerns are the following. First, the literature review is described as systematic but is incompletely reported, lacking a search strategy, a PRISMA flow diagram, and any account of screening, the number of reviewers, or how disagreements were resolved. Second, the interviews lack an explicit and robust qualitative design; no coding framework, inter-coder reliability, or interview guide is provided. Moreover, the inclusion of non-nurse participants in a nursing competency framework is not conceptually justified. Third, the Delphi process raises concerns of interpretation and transparency: the stopping criteria, the calculation of the expert authority coefficient and the basis for item changes are not clearly specified. Furthermore, the panel is small and skewed by geography and discipline, bearing on the framework's claimed national applicability. Fourth, the AHP weighting is markedly underspecified, with no rationale for the method and no account of who performed the pairwise judgments, how many respondents contributed, or how they were aggregated and checked for consistency.

Minor issues comprise a typographical error and low resolution in Figure 1, and a need for language editing to enhance text clarity.

Overall, this study is relevant as it addresses an important workforce question, and the resulting framework is a valuable contribution. However, the concerns identified are extensive and significant, affecting every methodological component of the study. Addressing them will require a genuinely thorough revision. I wish to emphasize that the authors should undertake a comprehensive reworking of the Methods and Results sections, substantially expanding the reporting of the literature review, the interviews, the Delphi process, and the AHP weighting. While demanding, these revisions appear achievable with the data already collected and do not, in my assessment, reflect irreparable design flaws.

My recommendation is therefore **major revision**, on the understanding that the revision required is considerable.
