# Supplementary material for: Development of a competency framework for public health nurses in China: A modified Delphi study
Source: PLoS One. 2026 Oct 30;21(10):e0361940. doi: 10.1371/journal.pone.0361940 (PMC13722293; doi:10.1371/journal.pone.0361940)
Supplement: Supplementary file 4 [file pone.0361940.s002.docx]

**S2 File. Detailed literature search strategies**

**PubMed:** (("nurses, public health"[MeSH Terms] OR "nurses, community health"[MeSH Terms] OR "public health nursing"[MeSH Terms] OR "community health nursing"[MeSH Terms]) OR ("public health nurs*"[Title/Abstract] OR "community health nurs*"[Title/Abstract] OR "community based nurs*"[Title/Abstract] OR "primary care nurs*"[Title/Abstract] OR "primary health care nurs*"[Title/Abstract]) OR "district nurs*"[Title/Abstract]) AND (("competenc*"[Title/Abstract] OR ("assessment"[Title/Abstract] AND "tool"[Title/Abstract]) OR "instrumentation"[MeSH Subheading] OR "instrument*"[Title/Abstract])), Filters: English, Humans.

**Embase:** ('community health nursing'/exp OR 'community health nurs*':ti,ab,kw OR 'community based nurs*':ti,ab,kw OR 'public health nurs*':ti,ab,kw OR 'primary care nurs*':ti,ab,kw OR 'primary health care nurs*':ti,ab,kw OR 'district nurs*':ti,ab,kw) AND (competenc*:ti,ab,kw OR (assessment:ti,ab,kw AND tool:ti,ab,kw) OR instrument*:ti,ab,kw) AND [english]/lim.

**Web of Science:** ((TS=("public health nurs*" or "community health nurs*" or "community based nurs*" or "primary care nurs*" or "primary health care nurs*" or "district nurs*")) AND TS=((competenc* or (assessment and tool) or instrument*)) AND LA=(English).

**CNKI：**	(TKA=“社区护士” + “公共卫生护士” + “基层护士” + “初级保健护士” + “社区护理” + “公共卫生护理” + “基层护理” + “初级保健护理”) AND (TKA=“能力” + “胜任力” + “评价指标” + “评估工具”)。

**Wanfang：**主题:(“社区护士” or “公共卫生护士”or “基层护士” or “初级保健护士”or “社区护理” or “公共卫生护理” or “基层护理” or “初级保健护理”) and 主题:(“能力” or “胜任力” or “评价指标” or “评估工具”)。

**VIP：**(M=“社区护士” OR M=“公共卫生护士” OR M=“基层护士” OR M=“初级保健护士” OR M=“社区护理” OR M=“公共卫生护理”OR M=“基层护理” OR M=“初级保健护理”) AND (M=“能力” OR M=“胜任力” OR M=“评价指标” OR M=“评估工具”)。

**Note:** The Chinese database search fields were retained as originally used. TKA in CNKI, Subject (主题) in Wanfang, and M in VIP correspond to searches covering the title, keywords, and abstract fields. The symbol “+” in CNKI is equivalent to the Boolean operator OR.
