# Supplementary material for: Development of a competency framework for public health nurses in China: A modified Delphi study
Source: PLoS One. 2026 Oct 30;21(10):e0361940. doi: 10.1371/journal.pone.0361940 (PMC13722293; doi:10.1371/journal.pone.0361940)
Supplement: Supplementary file 5 [file pone.0361940.s003.docx]

# S3 File. Semi-structured Interview Guide

[English Translation]

## Interview Guide for Community Physicians

1. What public health services are currently provided in your institution, and how are these services delivered?

2. What are your main work responsibilities in your current institution? How do you collaborate with nurses in the delivery of public health services?

3. In your work, what competencies of nursing colleagues contribute to effective public health service delivery?

4. What challenges have you encountered in collaborating with nurses?

5. What competencies do you expect nurses collaborating with you to have? What are the key elements of these competencies? Which competencies are particularly important?

6. From your perspective, please discuss your views and suggestions regarding the improvement of public health nursing competencies and the future development of community nurses.

## Interview Guide for Community Nurses

1. What public health services are currently provided in your institution, and how are these services delivered?

2. What are your main work responsibilities in your current institution?

3. In your work, what competencies do you possess that contribute to effective public health nursing practice?

4. What challenges have you encountered in your work?

5. If you were interviewing candidates for a public health nursing position, what competencies or qualities would you consider necessary for performing public health nursing work effectively? What are the key elements of these competencies? Which aspects would you consider most important?

6. From your perspective, please discuss your views and suggestions regarding the improvement of public health nursing competencies and the future development of community nurses.

## Interview Guide for Nursing Administrators

1. What public health services are currently provided in your institution, and how are these services delivered?

2. What are your main work responsibilities in your current institution? How many nurses are currently employed in your community health service team? Are human resources for basic medical care and basic public health services managed separately?

3. In your current work, what competencies do nurses engaged in public health nursing generally possess?

4. In your current work, which competencies of nurses engaged in public health nursing are relatively insufficient?

5. When assigning nurses to public health nursing positions, what competencies or qualities would you consider necessary for successful performance? What are the key elements of these competencies? What factors would you consider when making this assessment?

6. From your perspective, please discuss your views and suggestions regarding the improvement of public health nursing competencies and the future development of community nurses.
