# Supplementary material for: Development of a competency framework for public health nurses in China: A modified Delphi study
Source: PLoS One. 2026 Oct 30;21(10):e0361940. doi: 10.1371/journal.pone.0361940 (PMC13722293; doi:10.1371/journal.pone.0361940)
Supplement: Supplementary file 6 [file pone.0361940.s004.docx]

**S4 File. Consolidated Criteria for Reporting Qualitative Research (COREQ) Checklist**

| **Topic** | **Guide Questions/Description** | **Reported in Section** |
| --- | --- | --- |
| **Domain 1: Research team and reflexivity** |  |  |
| Personal characteristics |  |  |
| Interviewer/facilitator | Which author/s conducted the interview or focus group? | Methods: Semi-structured interviews |
| Credentials | What were the researcher’s credentials? E.g. PhD, MD | Methods: Semi-structured interviews |
| Occupation | What was their occupation at the time of the study? | Methods: Semi-structured interviews |
| Gender | Was the researcher male or female? | Not reported |
| Experience and training | What experience or training did the researcher have? | Methods: Semi-structured interviews |
| Relationship with participants |  |  |
| Relationship established | Was a relationship established prior to study commencement? | Methods: Semi-structured interviews |
| Participant knowledge of the interviewer | What did the participants know about the researcher? e.g. personal goals, reasons for doing the research | Methods: Semi-structured interviews |
| Interviewer characteristics | What characteristics were reported about the interviewer/facilitator? e.g. Bias, assumptions, reasons and interests in the research topic | Not reported |
| **Domain 2: Study design** |  |  |
| Theoretical framework |  |  |
| Methodological orientation and Theory | What methodological orientation was stated to underpin the study? e.g. grounded theory, discourse analysis, ethnography, phenomenology, content analysis | Methods: Semi-structured interviews |
| Participant selection |  |  |
| Sampling | How were participants selected? e.g. purposive, convenience, consecutive, snowball | Methods: Semi-structured interviews |
| Method of approach | How were participants approached? e.g. face-to-face, telephone, mail, email | Methods: Semi-structured interviews |
| Sample size | How many participants were in the study? | Results: Preliminary competency framework |
| Non-participation | How many people refused to participate or dropped out? Reasons? | Results: Preliminary competency framework |
| Setting |  |  |
| Setting of data collection | Where was the data collected? e.g. home, clinic, workplace | Methods: Semi-structured interviews |
| Presence of non-participants | Was anyone else present besides the participants and researchers? | Methods: Semi-structured interviews |
| Description of sample | What are the important characteristics of the sample? e.g. demographic data, date | Results: Preliminary competency framework |
| Data collection |  |  |
| Interview guide | Were questions, prompts, guides provided by the authors? Was it pilot tested? | Methods: Semi-structured interviews; S3 File |
| Repeat interviews | Were repeat interviews carried out? If yes, how many? | Not reported |
| Audio/visual recording | Did the research use audio or visual recording to collect the data? | Methods: Semi-structured interviews |
| Field notes | Were field notes made during and/or after the interview or focus group? | Methods: Semi-structured interviews |
| Duration | What was the duration of the interviews or focus group? | Results: Preliminary competency framework |
| Data saturation | Was data saturation discussed? | Methods: Semi-structured interviews |
| Transcripts returned | Were transcripts returned to participants for comment and/or correction? | Not reported |
| **Domain 3: analysis and findings** |  |  |
| Data analysis |  |  |
| Number of data coders | How many data coders coded the data? | Methods: Semi-structured interviews |
| Description of the coding tree | Did authors provide a description of the coding tree? | Not reported |
| Derivation of themes | Were themes identified in advance or derived from the data? | Methods: Semi-structured interviews |
| Software | What software, if applicable, was used to manage the data? | Methods: Semi-structured interviews |
| Participant checking | Did participants provide feedback on the findings? | Not reported |
| Reporting |  |  |
| Quotations presented | Were participant quotations presented to illustrate the themes/findings? Was each quotation identified? e.g. participant number | Not reported |
| Data and findings consistent | Was there consistency between the data presented and the findings? | Not reported |
| Clarity of major themes | Were major themes clearly presented in the findings? | Results: Preliminary competency framework |
| Clarity of minor themes | Is there a description of diverse cases or discussion of minor themes? | Not reported |
