# Supplementary material for: Development of a competency framework for public health nurses in China: A modified Delphi study
Source: PLoS One. 2026 Oct 30;21(10):e0361940. doi: 10.1371/journal.pone.0361940 (PMC13722293; doi:10.1371/journal.pone.0361940)
Supplement: Supplementary file 7 [file pone.0361940.s006.docx]

**S1 Table. Summary of** **competency elements identified from the literature review**

| **No.** | **Year** | **Country/Region** | **Organization/Authors** | **Study focus** | **Main competency elements identified** |
| --- | --- | --- | --- | --- | --- |
| 1 | 2021 | USA | Council on Linkages Between Academia and Public Health Practice [1] | Core Competencies for Public Health Professionals | 1. Data analytics and assessment skills; 2. Policy development and program planning skills; 3. Communication skills; 4. Health equity skills; 5. Community partnership skills; 6. Public health sciences skills; 7. Management and finance skills; 8. Leadership and systems thinking skills |
| 2 | 2019 | USA | Larsen et al. [2] | Tier 1 Core Competencies for Public Health Nurses | 1. Analytic and assessment skills; 2. Policy development and program planning skills; 3. Communication skills; 4. Cultural competency skills; 5. Community partnership skills; 6. Public health sciences skills; 7. Financial planning and management skills; 8. Leadership and systems thinking skills |
| 3 | 2018 | USA | Quad Council Coalition Competency Review Task Force [3] | Community/Public Health Nursing Competencies (Tier 1–3) | 1. Assessment and analytical skills; 2. Policy development and program planning skills; 3. Communication skills; 4. Cultural competency skills; 5. Community partnership skills; 6. Public health sciences skills; 7. Financial planning and management skills; 8. Leadership and systems thinking skills |
| 4 | 2014 | USA | Hewitt et al. [4] | Family Planning Public Health Nursing Competency Model | 1. Assessment/Evaluation; 2. Provide/Administer medications and contraceptives; 3. Education/Counseling |
| 5 | 2013 | USA | American Association of Colleges of Nursing (AACN) [5] | Baccalaureate Competencies and Curricular Guidelines for Public Health Nursing | 1. Liberal education for baccalaureate generalist nursing practice; 2. Basic organizational and systems leadership for quality care and patient safety; 3. Scholarship for evidence-based practice; 4. Information management and application of patient care technology; 5. Health care policy, finance, and regulatory environments; 6. Interprofessional communication and collaboration; 7. Clinical prevention and population health; 8. Professionalism and professional values; 9. Baccalaureate generalist nursing practice |
| 6 | 2013 | USA | Reckinger et al. [6] | Public Health Nursing Competency Instrument: Scale Reduction | 1. Assessment competencies; 2. Planning competencies; 3. Evaluation competencies; 4. Individual/family/community competencies; 5. Systems competencies; 6. Partnership/collaboration competencies |
| 7 | 2013 | Americas Region | Pan American Health Organization (PAHO)/World Health Organization (WHO) [7] | Regional Core Competency Framework for Public Health | 1. Health situation analysis; 2. Surveillance and control of risks and threats; 3. Health promotion and social participation; 4. Policy, planning, regulation and control; 5. Equitable access and quality of individual and public health services; 6. International/global health |
| 8 | 2011 | USA | Henry Street Consortium [8] | Population-Based Public Health Nursing Competency | 1. Applies the public health nursing process; 2. Utilizes basic epidemiological principles; 3. Collaboration; 4. Governmental public health system practice; 5. Legal scope of nursing practice; 6. Communication; 7. Caring relationships; 8. Commitment to social justice and public health principles; 9. Cultural acceptance and respect; 10. Holistic health assessment and intervention; 11. Leadership |
| 9 | 2010 | USA | Association of Community Health Nursing Educators (ACHNE) [9] | Essentials of Baccalaureate Nursing Education for Entry-Level Community/Public Health Nursing | 1. Core professional values: community/population as client; prevention; partnership; healthy environment; diversity. 2. Core knowledge and basic competencies: communication; epidemiology and biostatistics; community/population assessment; community/population planning; policy development; assurance; health promotion and risk reduction; illness and disease management; information and health care technology; environmental health; global health; human diversity; ethics and social justice; coordinator and manager role; emergency preparedness, response, and recovery. |
| 10 | 2008 | USA | Polivka et al. [10] | Public Health Nursing Competencies for Public Health Surge Events | 1. Preparedness; 2. Response; 3. Recovery |
| 11 | 2006 | USA | Cross et al. [11] | Public Health Nursing Competency Instrument | 1. Population-based assessment; 2. population-based planning; 3. implementation of interventions; 4. evaluation of processes and outcomes |
| 12 | 2006 | USA | Issel et al. [12] | Self-Reported Public Health Nursing Competency Scale | 1. Monitoring community health status; 2. Informing, educating, and empowering populations at risk; 3. Mobilizing community partnerships; 4. Policy and planning skills; 5. Enforcement of laws and regulations; 6. Linking people to services; 7. Ensuring a competent workforce; 8. Evaluating health services; 9. Researching innovative solutions |
| 13 | 2015 | Canada | Community Health Nurses of Canada (CHNC) [13] | Leadership Competencies for Public Health Practice in Canada | 1. Systems transformation; 2. Achieve results; 3. Lead self; 4. Engage others; 5. Develop coalition |
| 14 | 2014 | Canada | Canadian Association of Schools of Nursing (CASN) [14] | Entry-to-Practice Public Health Nursing Competencies for Undergraduate Nursing Education | 1. Public health sciences in nursing practice; 2. Population and community health assessment and analysis; 3. Population health planning, implementation, and evaluation; 4. Partnerships, collaboration and advocacy; 5. Communication in public health nursing |
| 15 | 2010 | Canada | Community Health Nurses of Canada [15] | Home Health Nursing Competencies | 1. Elements of Home Health Nursing; 2. Foundations of Home Health Nursing; 3. Quality and Professional Responsibility |
| 16 | 2009 | Canada | Community Health Nurses Association of Canada (CHNAC) [16] | Public Health Nursing Discipline Specific Competencies | 1. Public health and nursing sciences; 2. Assessment and analysis; 3. Policy and program planning, implementation and evaluation; 4. Partnerships, collaboration and advocacy; 5. Diversity and inclusiveness; 6. Communication; 7. Leadership; 8. Professional responsibility and accountability |
| 17 | 2008 | Canada | Public Health Agency of Canada (PHAC) [17] | Core Competencies for Public Health in Canada | 1. Attitudes and values; 2. Public health sciences; 3. Assessment and analysis; 4. Policy and program planning; 5. Implementation and evaluation; 6. Partnerships, collaboration and advocacy; 7. Diversity and inclusiveness; 8. Communication; 9. Leadership |
| 18 | 2017 | Japan | National Association of Public Health Nurse Education Institutions [18] | Public Health Nursing Education Model Core Curriculum (2017): Basic qualities and competencies required of public health nurses | 1. Professionalism; 2. Public health nursing knowledge and problem-solving ability; 3. Public health nursing practice competence; 4. Communication competence; 5. Collaboration competence; 6. Quality assurance of care and safety management; 7. Social trends and public health nursing activities; 8. Scientific inquiry; 9. Lifelong learning attitude |
| 19 | 2010 | Japan | Japan Academy of Community Health Nursing (JACHN) [19] | Public health nursing practice competencies | 1. Community health needs identification and planning; 2. Continued support and collaborative/systematic activities for community health promotion; 3. Community health crisis management; 4. Social resource development, systematization and policy-making for community health improvement; 5. Professional autonomy and continuous quality improvement. |
| 20 | 2022 | Singapore | National Nursing Academy (NNA) [20] | Community Nursing Competency Framework | 1. Person-centred care; 2. Population-based practice; 3. Professional development and leadership; 4. Improvement, innovation and research |
| 21 | 2018 | Europe | Association of Schools of Public Health in the European Region (ASPHER) [21] | ASPHER’s European List of Core Competences for the Public Health Professional | 1. Methods in public health; 2. Population health and its social, economic and political determinants; 3. Population health and its material, physical, radiological, chemical and biological environmental determinants; 4. Health policy, economics, organisational theory, leadership and management; 5. Health promotion, health protection and disease prevention; 6. Ethics |
| 22 | 2016 | Australia | Council of Academic Public Health Institutions Australia (CAPHIA) [22] | Foundation Competencies for Public Health Graduates in Australia | 1. Health Monitoring and Surveillance; 2. Disease Prevention and Control; 3. Health Protection; 4. Health Promotion; 5. Health Policy, Planning and Management; 6. Evidence-Based Professional Population Health Practice |
| 23 | 2005 | UK | Fiona Irvine [23] | Health promotion competencies for district nurses | 1. Knowledge: Health and its determinants; Ill health; Accessing resources and data; Target, guidelines, policy and practice; Lifestyle and behaviour; Supporting evidence; Theory and practice of health promotion; Teamworking. 2. Attitudes: Empowerment; Holism; Respect for individuals; Proactive stance; Commitment to health promotion; Self-awareness; Possible successes and limitations of health promotion. 3. Skills: Teamworking; Community working; Personal skills; Opportunism; Time management; Client group identification; Communication; Information gathering and interpretation. |
| 24 | 2021 | China | Lin Y et al. [24] | Evaluation indicators for public health emergency response competencies among community nurses | 1. Emergency prevention; 2. Emergency preparedness; 3. Emergency response; 4. Emergency recovery. |
| 25 | 2020 | China | Liang SX [25] | Core competency framework for basic public health service personnel | 1. Professional knowledge (public health and preventive medicine knowledge, clinical medicine knowledge, health care knowledge, and interdisciplinary knowledge); 2. Practical skills (community health surveillance, community health needs assessment, policy and intervention development, implementation and evaluation, health management, infectious disease and public health emergency reporting and response, health supervision and coordination, information technology application, communication, partnership development, management and leadership, and basic scientific research); 3. Concepts and values (service philosophy, lifelong learning philosophy, professional attributes, and professional spirit). |
| 26 | 2018 | China | Li Q et al. [26] | Competency evaluation indicator system for community nurses | 1. Knowledge (community nursing knowledge, related knowledge of community nursing, and basic public health knowledge); 2. Skills (basic community nursing skills and specialized community nursing skills); 3. Competencies (critical thinking, interpersonal communication, management, research, teaching, and self-directed learning and professional development); 4. Characteristics and qualities (professional ethics, psychological attributes, and physical attributes). |
| 27 | 2017 | China | Cao J et al. [27] | Core competency evaluation indicator system for community nurses in elderly care | 1. Elderly health assessment and planning; 2. Nursing technical practice; 3. Education and guidance; 4. Service quality improvement; 5. Interpersonal communication; 6. Professional development; 7. Legal and ethical practice. |
| 28 | 2017 | China | Li GH et al. [28] | Core competencies in public health | 1. Public health ethics and law; 2. Public health professional competency; 3. Policy development and implementation; 4. Leadership. |
| 29 | 2017 | China | Xiang SM et al. [29] | Health education competency assessment scale for community nurses | 1. Assessment; 2. Planning; 3. Implementation; 4. Evaluation. |
| 30 | 2016 | China | Zhai YP et al. [30] | Core competency evaluation indicator system for generalist nurses | 1. Communication; 2. Disease care; 3. Health promotion; 4. Ethical and legal practice; 5. Organizational management; 6. Critical thinking; 7. Professional development. |
| 31 | 2016 | China | Wang DY et al. [31] | Public health emergency response competency questionnaire for community nurses | 1. Emergency response knowledge; 2. Comprehensive competency; 3. First-aid skills. |
| 32 | 2016 | China | Guo YJ et al. [32] | Core competency evaluation indicator system for public health personnel | 1. Professional competency; 2. Research competency; 3. Comprehensive competency. |
| 33 | 2015 | China | General Office of the National Health and Family Planning Commission [33] | Community health service quality evaluation indicator system | 1. Service capacity (medical services; public health services: community health diagnosis, health records, health education, immunization, health management for key populations, health management for priority diseases, public services, family planning technical services, and prevention and control of birth defects); 2. Service quality; 3. Institutional management; 4. Support conditions. |
| 34 | 2015 | China | Lou Y et al. [34] | Core competency evaluation indicator system for community nurses | 1. Community nursing practice; 2. Critical thinking; 3. Management; 4. Communication and coordination; 5. Professional development; 6. Legal and ethical practice. |
| 35 | 2015 | China | Wu H et al. [35] | Self-assessment questionnaire on home care competencies for community nurses | 1. Home care practice; 2. Critical thinking; 3. Management and organization; 4. Interpersonal communication; 5. Self-development. |
| 36 | 2013 | China | Huo SP et al. [36] | Comprehensive professional competency scale for community nurses | 1. Comprehensive nursing; 2. Education, teaching, and guidance; 3. Research; 4. Interpersonal communication; 5. Self-development; 6. Coordination and management; 7. Emergency response. |
| 37 | 2012 | China | Chen YY et al [37] | Core competency evaluation indicator system for community nurses | 1. Community nursing practice; 2. Communication and coordination; 3. Legal and ethical practice; 4. Critical thinking; 5. Professional development. |
| 38 | 2012 | China | Guo L et al. [38] | Questionnaire on community nursing service delivery by community nurses | 1. Technical nursing services; 2. Chronic disease health management; 3. Community coordination; 4. Health management for women, children, and adolescents; 5. Health education; 6. Other services. |
| 39 | 2011 | China | Qu LX [39] | Core competency assessment questionnaire for community nurses | 1. Critical thinking; 2. Interpersonal communication; 3. Assessment and intervention; 4. Leadership and management; 5. Legal and ethical practice; 6. Professional development. |
| 40 | 2010 | China | Yang YN et al. [40] | Disaster response competency evaluation indicator system for community nurses | 1. Knowledge base; 2. Crisis assessment; 3. Emergency response; 4. Psychological stress management; 5. Post-disaster disease prevention; 6. Personal attributes. |

**Reference**

1. Public Health Foundation. Core competencies for public health professionals [Internet]. Washington (DC): Public Health Foundation; 2021 Oct 21 [cited 2023 Mar 16]. Available from: http://www.phf.org/resourcestools/pages/core_public_health_competencies.aspx

2. Larsen R, Reif L. Leveling the core competencies of public health nursing to evaluate senior baccalaureate nursing students. Public Health Nurs. 2019;36(5):744-751.

3. Quad Council Coalition. Community/Public Health Nursing (C/PHN) competencies [Internet]. Quad Council Coalition; 2018 [cited 2022 Sep 1]. Available from: https://www.cphno.org/wp-content/uploads/2020/08/QCC-C-PHN-COMPETENCIES-Approved_2018.05.04_Final-002.pdf

4. Hewitt CM, Roye C, Gebbie KM. Core competency model for the family planning public health nurse. Public Health Nurs. 2014;31(5):472-479.

5. American Association of Colleges of Nursing. Recommended baccalaureate competencies and curricular guidelines for public health nursing [Internet]. Washington (DC): American Association of Colleges of Nursing; 2013 [cited 2023 Mar 16]. Available from: https://www.aacnnursing.org/Portals/0/PDFs/Teaching-Resources/BSN-Curriculum-Guide.pdf

6. Reckinger D, Cross S, Block DE, Josten L, Savik K. Public health nursing competency instrument: scale reduction and reliability of factors. Public Health Nurs. 2013;30(6):566-574.

7. Pan American Health Organization. Core competencies for public health: a regional framework for the Americas [Internet]. Washington (DC): Pan American Health Organization; 2013 [cited 2023 Mar 16]. Available from: https://iris.paho.org/server/api/core/bitstreams/9c982c62-c447-489f-9ddc-b90aab8a92ba/content

8. Schaffer MA, Cross S, Keller LO, Nelson P, Schoon PM, Henton P. The Henry Street Consortium population-based competencies for educating public health nursing students. Public Health Nurs. 2011;28(1):78-90.

9. Education Committee of the Association of Community Health Nurse Educators. Essentials of baccalaureate nursing education for entry-level community/public health nursing. Public Health Nurs. 2010;27(4):371-382.

10. Polivka BJ, Stanley SA, Gordon D, Taulbee K, Kieffer G, McCorkle SM. Public health nursing competencies for public health surge events. Public Health Nurs. 2008;25(2):159-165.

11. Cross S, Block D, Josten L, Reckinger D, Olson Keller L, Strohschein S, et al. Development of the public health nursing competency instrument. Public Health Nurs. 2006;23(2):108-114.

12. Issel LM, Baldwin KA, Lyons RL, Madamala K. Self-reported competency of public health nurses and faculty in Illinois. Public Health Nurs. 2006;23(2):168-177.

13. Community Health Nurses of Canada. Leadership competencies for public health practice in Canada [Internet]. Canada: Community Health Nurses of Canada; 2015 [cited 2023 Mar 16]. Available from: https://fliphtml5.com/fghr/vrgv/basic

14. Canadian Association of Schools of Nursing. Entry-to-practice public health nursing competencies for undergraduate nursing education [Internet]. Ottawa (Canada): Canadian Association of Schools of Nursing; 2014 [cited 2022 Sep 13]. Available from: https://casn.ca/wp-content/uploads/2014/12/FINALpublichealthcompeENforweb.pdf

15. Community Health Nurses of Canada. Home health nursing competencies. Version 1.0 [Internet]. Canada: Community Health Nurses of Canada; 2010 [cited 2022 Sep 13]. Available from: https://www.chnc.ca/en/competencies

16. Community Health Nurses of Canada. Public health nursing discipline specific competencies. Version 1.0 [Internet]. Canada: Community Health Nurses of Canada; 2009 May [cited 2022 Aug 29]. Available from: https://www.chnc.ca/en/competencies

17. Public Health Agency of Canada. Core competencies for public health in Canada: release 1.0 [Internet]. Ottawa (Canada): Public Health Agency of Canada; 2008 [cited 2023 Mar 16]. Available from: https://www.canada.ca/content/dam/phac-aspc/documents/services/public-health-practice/skills-online/core-competencies-public-health-canada/cc-manual-eng090407.pdf

18. National Association of Public Health Nurse Educational Institutions. Public health nursing education model core curriculum [Internet]. Japan: National Association of Public Health Nurse Educational Institutions; 2017 [cited 2023 Mar 16]. Available from: https://zenhokyo.jp/others/doc/201911-curriculum-kentou_6.pdf Japanese.

19. Japan Academy of Community Health Nursing. JACHN Newsletter No. 10 [Internet]. Japan: Japan Academy of Community Health Nursing; 2010 [cited 2022 Sep 12]. Available from: http://jachn.umin.jp/newsletter/10.NewsFromJACHN2010.pdf Japanese.

20. National Nursing Academy. Community nursing competency framework [Internet]. Singapore: National Nursing Academy; 2022 [cited 2023 Mar 9]. Available from: https://www.nna.gov.sg/resources-tools/nursing-competency-framework/community-nursing-competency-framework

21. Association of Schools of Public Health in the European Region. ASPHER's European list of core competences for the public health professional. Scand J Public Health. 2018;46(23 Suppl):1-52.

22. Council of Academic Public Health Institutions Australasia. Foundation competencies for public health graduates in Australia. 2nd ed [Internet]. Australia: Council of Academic Public Health Institutions Australasia; 2016 [cited 2023 Mar 16]. Available from: https://caphia.com.au/competencies/

23. Irvine F. Exploring district nursing competencies in health promotion: the use of the Delphi technique. J Clin Nurs. 2005;14(8):965-975.

24. Lin Y, Zhu XH, Chen Y. Construction of an emergency rescue competence index system for public health emergency among community nurses. Chin J Nurs. 2021;56(8):1158-1164. Chinese.

25. Liang SX. Research on the core competence building of basic public health service workers in primary health care sectors in Western China [dissertation]. Chongqing (China): Army Medical University of the Chinese People's Liberation Army; 2020. Chinese.

26. Li Q, Fu LM, Fang LY, Deng H, Lai D. Construction of evaluation index system for competency of community nurses. Chin Nurs Res. 2018;32(24):3866-3869. Chinese.

27. Cao J, Wang JQ, Liang Y, Chen Y. Development of gerontology community nurse competencies. J Nurs Sci. 2017;32(4):93-96. Chinese.

28. Li GH, Zou Z, Zhang L, Wu F, Zheng ZJ. The development of public health core competence and public health education. Soft Sci Health. 2017;31(10):43-45,51. Chinese.

29. Xiang SM, Wang DY, Zhang WL, Xu XM, Fang JM. Investigation on Health Education Ability and Its Influencing Factors of Community Nurses in Wenzhou City. Nurs J Chin PLA. 2017;34(24):20-23. Chinese.

30. Zhai YP, Xu JJ. Construction of evaluation index system of core competence of general nurses. Chin Nurs Res. 2016;30(5):533-539. Chinese.

31. Wang DY, Zhang WL, Xiang SM, Xu XM, Fang JM. Investigation on community nurses' emergency preparedness in public health emergency in Wenzhou city. J Nurs Sci. 2016;31(4):82-84. Chinese.

32. Guo YJ, Yu DD, Huang XM, Yu C, Cheng SY. Using Delphi method to establish evaluation indicator system for core competence of public health professionals. Chin J Public Health Manag. 2016;32(6):771-773. Chinese.

33. Department of Primary Health, National Health and Family Planning Commission of the People's Republic of China. Notice on carrying out the community health service improvement project [Internet]. Beijing (China): National Health and Family Planning Commission of the People's Republic of China; 2015 Nov 25 [cited 2022 Feb 13]. Available from: http://www.nhc.gov.cn/jws/s3581r/201511/c1100cd1890848eabf0a899539eaba13.shtml Chinese.

34. Lou Y, Ma XQ, Song YQ. The development of a core competence evaluation index system for community nurses. J Nurs Adm. 2015;15(3):153-155. Chinese.

35. Wu H, Shi ZX, Huang LK, Wang XC, Zhang T, Gu XL. Current situation investigation and analysis of community nurse′s home care ability in Yingze District Taiyuan. Chin J Mod Nurs. 2015;(30):3635-3639. Chinese.

36. Huo SP, Ke YX, Li J, Zou SS. Development and reliability and validity testing of a professional comprehensive competency scale for community nurses. J Nurs (China). 2013;(12):71-74. Chinese.

37. Chen YY, Yu LZ. Study on construction of core competency evaluation index of community nurses and its reliability and validity evaluation. Chin Nurs Res. 2012;26(30):2873-2874,2879. Chinese.

38. Guo L, Liu SZ. Study on Present Situation of Nursing Services Provided by Community Health Nurses. Chin Gen Pract. 2012;15(8):929-932. Chinese.

39. Qu LX. Current status and training strategies of core competencies among community nurses in Changchun [master's thesis]. Changchun (China): Jilin University; 2011. Chinese.

40. Yang YN, Luo Y. Study on construction of evaluation index system for disaster preparedness and coping capacity of community nurses. Chin Nurs Res. 2010;24(10):855-858. Chinese.
