# Supplementary material for: Development of a competency framework for public health nurses in China: A modified Delphi study
Source: PLoS One. 2026 Oct 30;21(10):e0361940. doi: 10.1371/journal.pone.0361940 (PMC13722293; doi:10.1371/journal.pone.0361940)
Supplement: Supplementary file 8 [file pone.0361940.s007.docx]

**S5 File. Expert Consultation Questionnaire for the Development of a Public Health Nursing**

**Competency Framework (Round 1)**

[English Translation]

Dear Expert,

Thank you for taking the time to participate in this expert consultation.

With the increasing emphasis on shifting the focus of health care toward prevention, China has been making substantial efforts to strengthen its public health system and workforce. Public health nursing personnel constitute an important part of the community public health workforce and play a critical role in protecting and promoting population health and delivering primary-level public health services. However, public health nursing in China remains at an early and exploratory stage, with gaps in professional competencies that may hinder the provision of high-quality public health nursing services. In addition, targeted educational programs for preparing public health nursing specialists remain limited, and the competencies required of public health nursing personnel to meet growing population health needs have yet to be clearly defined.

Against this background, we are conducting a study to develop a competency framework for public health nursing. The study aims to identify the competencies and professional attributes required for public health nursing practice, with the goal of promoting the professionalization, standardization, and quality of public health nursing practice in China and contributing to the development of the public health workforce.

In the preliminary stage of this study, guided by the Minnesota Public Health Interventions Wheel, China’s Basic Public Health Service framework, and the International Council of Nurses (ICN) Framework of Competencies for the Generalist Nurse, we developed a preliminary competency framework based on a comprehensive literature review, job analysis, and semi-structured interviews. The framework will be further refined through consultation with a broader panel of experts. Given your extensive expertise and practical experience in this field, we sincerely invite you to participate as an expert in this consultation. Your comments and suggestions will provide an important basis for refining the competency framework.

To facilitate the progress of the study, we kindly ask you to complete and return this round of the questionnaire within one week. If you have any questions or require further information, please feel free to contact us by telephone or email using the contact details below.

Thank you again for your valuable time and contribution. We wish you good health and every success in your work.

Sincerely,

XXX

Tel: XXXX

E-mail: XXX

**Part I. Conceptual Definitions**

1. Public health nurse (PHN): A nurse working in primary health care settings who undertakes public health nursing responsibilities, primarily involving prevention, health maintenance, health education, and family planning services.

2. Competency: The systematic integration of knowledge, skills, judgment, and personal attributes (including attitudes, values, and beliefs) required for professionals to fulfill their role responsibilities and achieve desired practice outcomes.

**Part II. Expert Consultation on the Public Health Nursing Competency Framework**

**Instructions**

1. Tables 1–3 present the proposed domains, subdomains, and competencies of the Public Health Nursing Competency Framework, with operational definitions provided for the competencies. We invite your comments on the inclusion, wording, and importance of each item.

2. The importance of each item is rated using a 5-point Likert scale: 5 = very important, 4 = important, 3 = moderately important, 2 = slightly important, and 1 = not important. Please rate the importance of each item by placing a “√” in the corresponding column or entering the corresponding score. Please select only one response for each item.

3. If you consider the wording of an item inaccurate or believe that an item should be removed, please provide your suggested revision or deletion in the “Comments and suggestions” column. If you believe that any important items have been omitted, please add them in the “Additional items” section and rate their importance using the same scale. Please provide a response for every item.

**Table 1. Expert Consultation on the Domains of the Public Health Nursing Competency Framework**

| **Domains** | **Importance rating** | | | | | **Comments and suggestions** |
| --- | --- | --- | --- | --- | --- | --- |
|  | **5** | **4** | **3** | **2** | **1** |  |
| **1. Community surveillance and assessment** |  |  |  |  |  |  |
| **2. Health education and health promotion** |  |  |  |  |  |  |
| **3. Health management** |  |  |  |  |  |  |
| **4. Community public health emergency management** |  |  |  |  |  |  |
| **5. Partnership development and maintenance** |  |  |  |  |  |  |
| **6. Professionalism and professional development** |  |  |  |  |  |  |
| **Additional items** |  |  |  |  |  |  |

**Table 2. Expert Consultation on the Subdomains of the Public Health Nursing Competency Framework**

| **Domains** | **Subdomains** | **Importance rating** | | | | | **Comments and suggestions** |
| --- | --- | --- | --- | --- | --- | --- | --- |
|  |  | **5** | **4** | **3** | **2** | **1** |  |
| **1. Community surveillance and assessment** | **1.1 Community health surveillance** |  |  |  |  |  |  |
|  | **1.2 Community nursing assessment and analysis** |  |  |  |  |  |  |
| **Additional items** |  |  |  |  |  |  |  |
| **2. Health education and health promotion** | **2.1 Health education and health promotion program management** |  |  |  |  |  |  |
|  | **2.2 Health communication** |  |  |  |  |  |  |
| **Additional items** |  |  |  |  |  |  |  |
| **3. Health management** | **3.1 Health information and health record management** |  |  |  |  |  |  |
|  | **3.2 Population-focused health management** |  |  |  |  |  |  |
|  | **3.3 Condition-specific health management** |  |  |  |  |  |  |
|  | **3.4 Setting-specific health management** |  |  |  |  |  |  |
|  | **3.5 TCM health management** |  |  |  |  |  |  |
| **Additional items** |  |  |  |  |  |  |  |
| **4. Community public health emergency management** | **4.1 Risk management** |  |  |  |  |  |  |
|  | **4.2 Emergency response** |  |  |  |  |  |  |
|  | **4.3 Recovery** |  |  |  |  |  |  |
| **Additional items** |  |  |  |  |  |  |  |
| **5. Partnership development and maintenance** | **5.1 Teamwork** |  |  |  |  |  |  |
|  | **5.2 Communication and coordination** |  |  |  |  |  |  |
| **Additional items** |  |  |  |  |  |  |  |
| **6. Professionalism and professional development** | **6.1 Professionalism** |  |  |  |  |  |  |
|  | **6.2 Professional development** |  |  |  |  |  |  |
| **Additional items** |  |  |  |  |  |  |  |

**Table 3. Expert Consultation on the Competencies of the Public Health Nursing Competency Framework**

| **Domains** | **Subdomains** | **Competencies and operational definitions** | **Importance rating** | | | | | **Comments and suggestions** |
| --- | --- | --- | --- | --- | --- | --- | --- | --- |
|  |  |  | **5** | **4** | **3** | **2** | **1** |  |
| **1. Community surveillance and assessment** | **1.1 Community health surveillance** | **1.1.1 Knowledge of health surveillance:** Knowledge of core concepts and terminology in epidemiology, including commonly used measures of disease frequency, intensity and distribution; and knowledge of basic hygiene requirements for living, food, and school environments, including drinking water hygiene standards and the concepts, classification, and identification of food contamination and foodborne diseases. |  |  |  |  |  |  |
|  |  | **1.1.2 Ability to collect health surveillance data:** The ability to systematically and continuously collect and manage health surveillance data on infectious diseases in the community (e.g., COVID-19, tuberculosis, HIV/AIDS, syphilis, hepatitis B), prevalent NCDs (e.g., type 2 diabetes, hypertension, malignant tumors), and community health hazards (e.g., food and drinking water safety risks, common school health problems and related risk factors, and reproductive health hazards). |  |  |  |  |  |  |
|  |  | **1.1.3 Ability to report and communicate health surveillance information:** The ability to report surveillance data to public health authorities and communicate essential findings to community residents. |  |  |  |  |  |  |
|  | **Additional items** |  |  |  |  |  |  |  |
|  | **1.2 Community nursing assessment and analysis** | **1.2.1 Ability to conduct comprehensive community nursing assessments:** The ability to conduct systematic community health assessments by collecting and integrating multi-source data on community demographics, environmental conditions, social systems, and population health status. |  |  |  |  |  |  |
|  |  | **1.2.2 Ability to identify health needs:** The ability to comprehensively and systematically identify existing and potential health needs within the community and anticipate future health needs. |  |  |  |  |  |  |
|  |  | **1.2.3 Ability to analyze health data:** The ability to interpret and compare data obtained from community nursing assessments to analyze community health problems and health needs. |  |  |  |  |  |  |
|  |  | **1.2.4 Ability to develop community nursing diagnoses:** The ability to synthesize data from comprehensive community assessments and available community health resources to develop community nursing diagnoses, identify priority health problems, and guide the planning of public health nursing interventions. |  |  |  |  |  |  |
|  | **Additional items** |  |  |  |  |  |  |  |
| **2. Health education and health promotion** | **2.1 Health education and health promotion program management** | **2.1.1 Ability to design health education and health promotion programs:** The ability to design targeted health education and health promotion programs based on community assessment data and informed by behavior change theories. |  |  |  |  |  |  |
|  |  | **2.1.2 Ability to implement health education and health promotion programs:** The ability to deliver targeted community health education through multiple approaches, including popular science materials, public health consultations, health education sessions, and individualized guidance. |  |  |  |  |  |  |
|  |  | **2.1.3 Ability to evaluate health education and health promotion programs:** The ability to evaluate health education and health promotion programs across multiple dimensions, including reach, acceptability, effectiveness, efficiency, and equity. |  |  |  |  |  |  |
|  | **Additional items** |  |  |  |  |  |  |  |
|  | **2.2 Health communication** | **2.2.1 Ability to analyze the context for health communication:** The ability to identify factors affecting health communication within the community, including the target audience’s literacy level, language, cultural background, and cognitive abilities, and determine communication channels accessible to and used by the target audience, such as social media and mass media. |  |  |  |  |  |  |
|  |  | **2.2.2 Ability to develop communication strategies:** The ability to apply communication theories and models to develop health communication strategies based on desired communication outcomes, such as raising awareness, promoting behavior change, and communicating health risks. |  |  |  |  |  |  |
|  |  | **2.2.3 Ability to select communication channels:** The ability to distinguish the advantages and disadvantages of different health communication channels, such as social media, health education sessions, peer communication, mass media, and mobile applications, and select the channels most appropriate for the target audience and the health information being communicated. |  |  |  |  |  |  |
|  |  | **2.2.4 Ability to design, develop, and use communication materials:** The ability to participate in the design, development, and use of health communication materials and effectively communicate various types of health information through selected channels and strategies. |  |  |  |  |  |  |
|  | **Additional items** |  |  |  |  |  |  |  |
| **3. Health management** | **3.1 Health information and health record management** | **3.1.1 Ability to use health information systems:** The ability to proficiently use public health information management systems. |  |  |  |  |  |  |
|  |  | **3.1.2 Ability to collect health information:** The ability to comprehensively, objectively, and accurately collect health information from service recipients and perform basic analysis and interpretation of the collected information. |  |  |  |  |  |  |
|  |  | **3.1.3 Ability to manage health records:** The ability to establish and manage residents’ health records in a standardized manner and dynamically update and maintain contents. |  |  |  |  |  |  |
|  | **Additional items** |  |  |  |  |  |  |  |
|  | **3.2 Population-focused health management** | **3.2.1 Ability to manage the health of children aged 0-6 years:** The ability to deliver comprehensive health management services for children aged 0-6 years, including newborn home visits, routine health checkups, immunization, and caregiver counseling on growth and development, nutrition, physical activity, and disease and injury prevention. |  |  |  |  |  |  |
|  |  | **3.2.2 Ability to manage the health of school-age children and adolescents:** The ability to provide health guidance for school-age children and adolescents on nutrition and diet, physical activity, mental health, and disease prevention. |  |  |  |  |  |  |
|  |  | **3.2.3 Ability to manage women’s health:** The ability to assess women’s health status across key life stages (e.g., premarital, perinatal, and perimenopausal periods) and provide tailored guidance on lifestyle, sexual and reproductive health, and mental well-being. |  |  |  |  |  |  |
|  |  | **3.2.4 Ability to manage older adults’ health:** The ability to assess the lifestyle and health status of older adults and provide tailored health guidance on nutrition and diet, physical activity, safety and injury prevention, and self-care. |  |  |  |  |  |  |
|  | **Additional items** |  |  |  |  |  |  |  |
|  | **3.3 Condition-specific health management** | **3.3.1 Ability to manage the health of people with NCDs:** The ability to conduct regular follow-up and routine monitoring, deliver nursing interventions, and provide self-management support for people with common chronic NCDs (e.g., hypertension and type 2 diabetes) to control risk factors and prevent complications. |  |  |  |  |  |  |
|  |  | **3.3.2 Ability to manage the health of people with infectious diseases:** The ability to provide referral, home visits (as appropriate), treatment adherence support, and follow-up for people with infectious diseases (e.g., tuberculosis and HIV), and deliver health education on prevention and control. |  |  |  |  |  |  |
|  |  | **3.3.3 Ability to manage the health of people with mental disorders:** The ability to provide information management, follow-up assessments, stratified interventions, and health examinations for patients with severe mental disorders, and provide guidance on daily living skills and psychological rehabilitation. |  |  |  |  |  |  |
|  | **Additional items** |  |  |  |  |  |  |  |
|  | **3.4 Setting-specific health management** | **3.4.1 Ability to manage health in school settings:** The ability to collect and manage students’ health information, conduct systematic health assessments, and provide guidance and health education on growth and development, nutrition, mental health, and sexual health. |  |  |  |  |  |  |
|  |  | **3.4.2 Ability to manage health in workplace settings:** The ability to contribute to occupational health surveillance and provide health education and counseling to workers on self-care, hazard identification, and injury prevention. |  |  |  |  |  |  |
|  | **Additional items** |  |  |  |  |  |  |  |
|  | **3.5 TCM health management** | **3.5.1 Ability to perform TCM constitution identification:** The ability to identify an individual’s body constitution according to the classification criteria for traditional Chinese medicine (TCM) body constitution. |  |  |  |  |  |  |
|  |  | **3.5.2 Ability to provide TCM-based health guidance:** The ability to provide TCM health care guidance tailored to different body constitutions, including dietary regulation, daily routine adjustment, physical activity recommendations, and emotional regulation, and offer basic instruction on commonly used meridians and acupoints. |  |  |  |  |  |  |
|  |  | **3.5.3 Ability to provide TCM nursing techniques:** Knowledge of the procedures and precautions for TCM nursing techniques, including moxibustion, cupping, hot compress therapy, and auricular acupressure with seeds. |  |  |  |  |  |  |
|  | **Additional items** |  |  |  |  |  |  |  |
| **4. Community public health emergency management** | **4.1 Risk management** | **4.1.1 Ability to conduct community risk assessments:** The ability to contribute to community risk assessments for infectious disease outbreaks and public health emergencies, including assessments of environmental hazards, population exposures, and community preparedness and response capacity. |  |  |  |  |  |  |
|  |  | **4.1.2 Ability to develop emergency response plans:** The ability to contribute to the development, review, and updating of emergency response plans for infectious diseases and public health emergencies. |  |  |  |  |  |  |
|  |  | **4.1.3 Ability to provide community education on emergency management:** The ability to deliver community education to residents on emergency preparedness, emergency response, and self-help and mutual aid skills. |  |  |  |  |  |  |
|  | **Additional items** |  |  |  |  |  |  |  |
|  | **4.2 Emergency response** | **4.2.1 Ability to report emergency information:** The ability to report infectious disease events and public health emergencies accurately and promptly, in accordance with prescribed timelines, channels, and procedures. |  |  |  |  |  |  |
|  |  | **4.2.2 Ability to implement public health emergency response measures and deliver emergency medical care:** The ability to contribute to implementing emergency response measures (e.g., isolation, medical observation, emergency vaccination, and chemoprophylaxis when indicated), and providing emergency medical care (e.g., pre-screening, triage, first aid, and referral). |  |  |  |  |  |  |
|  |  | **4.2.3 Ability to conduct epidemiological investigations:** The ability to contribute to community-based field epidemiologic investigations, including case investigation, contact tracing, and data collection. |  |  |  |  |  |  |
|  |  | **4.2.4 Ability to collect clinical specimens:** The ability to contribute to the safe collection, labeling, storage, and transport of clinical specimens in accordance with standard protocols. |  |  |  |  |  |  |
|  |  | **4.2.5 Ability to support containment and environmental decontamination measures in outbreak-affected areas:** The ability to contribute to the implementation of containment measures and environmental cleaning and disinfection in outbreak-affected areas. |  |  |  |  |  |  |
|  |  | **4.2.6 Ability to guide the implementation of community emergency protective measures:** The ability to guide community organizations and residents in the appropriate use of emergency medications and protective equipment and in implementing emergency response and self-help measures. |  |  |  |  |  |  |
|  | **Additional items** |  |  |  |  |  |  |  |
|  | **4.3 Recovery** | **4.3.1 Ability to conduct post-event evaluation:** The ability to conduct post-event evaluation of responses to infectious diseases and public health emergencies, to synthesize lessons learned, and to further improve emergency response plans. |  |  |  |  |  |  |
|  |  | **4.3.2 Ability to manage recovery-phase health issues:** The ability to mobilize partners to manage the health issues of priority populations during the recovery phase of a public health emergency, addressing both physical rehabilitation needs and psychological recovery support. |  |  |  |  |  |  |
|  | **Additional items** |  |  |  |  |  |  |  |
| **5. Partnership development and maintenance** | **5.1 Teamwork** | **5.1.1 Ability to establish and sustain collaboration with external community partners:** The ability to proactively identify, engage, and sustain partnerships with external stakeholders (e.g., government and health authorities, public health agencies, and other healthcare organizations) to secure support and resources for community public health initiatives. |  |  |  |  |  |  |
|  |  | **5.1.2 Ability to collaborate within the community health team:** The ability to establish and sustain effective interprofessional collaboration within the community health team. |  |  |  |  |  |  |
|  |  | **5.1.3 Ability to collaborate with community residents:** The ability to encourage volunteers and community organizations to participate in community public health activities and provide relevant training for them. |  |  |  |  |  |  |
|  | **Additional items** |  |  |  |  |  |  |  |
|  | **5.2 Communication and coordination** | **5.2.1 Ability to conduct effective communication:** The ability to communicate effectively by applying active listening and providing clear, timely, and empathetic responses to facilitate mutual understanding and build rapport. |  |  |  |  |  |  |
|  |  | **5.2.2 Ability to align stakeholders:** The ability to align and coordinate multisectoral stakeholders using team-building, negotiation, conflict management, and facilitation skills. |  |  |  |  |  |  |
|  | **Additional items** |  |  |  |  |  |  |  |
| **6. Professionalism and professional development** | **6.1 Professionalism** | **6.1.1 Professional responsibility:** The ability to demonstrate commitment to public health nursing and a strong sense of professional responsibility. |  |  |  |  |  |  |
|  |  | **6.1.2 Legal literacy:** The ability to apply relevant laws, regulations, and policies to ensure compliance in public health nursing practice. |  |  |  |  |  |  |
|  |  | **6.1.3 Ethical literacy:** The ability to apply ethical principles (e.g., utility, respect, and health equity) to address ethical issues in public health nursing practice. |  |  |  |  |  |  |
|  | **Additional items** |  |  |  |  |  |  |  |
|  | **6.2 Professional development** | **6.2.1 Ability to engage in self-directed learning:** The ability to engage in self-directed learning to maintain and enhance public health nursing knowledge and skills through multiple channels, including books, scholarly literature, online resources, academic exchanges, and continuing education. |  |  |  |  |  |  |
|  |  | **6.2.2 Ability to teach and mentor others:** The ability to educate and mentor students and novice nurses using appropriate pedagogical approaches. |  |  |  |  |  |  |
|  |  | **6.2.3 Ability to conduct continuous quality improvement:** The ability to conduct continuous quality improvement through a systematic process of data-driven gap identification, implementation of improvement strategies, and outcome monitoring. |  |  |  |  |  |  |
|  |  | **6.2.4 Ability to contribute to public health research projects:** The ability to participate in community public health research projects, write and publish research papers in the field, and facilitate the translation of research findings into community public health interventions. |  |  |  |  |  |  |
|  | **Additional items** |  |  |  |  |  |  |  |

**Part III. Expert Information**

**Instructions**

Please provide your basic information and self-assess the basis for your judgments and your familiarity with the consultation content by placing a “√” in the appropriate box.

**Table 4. Expert Demographic Information**

| Name |  | Gender |  | Age |  |
| --- | --- | --- | --- | --- | --- |
| Highest educational qualification |  | Professional title |  | Administrative position |  |
| Institution |  | | Field of expertise |  | |
| Years of professional experience |  | | | | |

**Table 5. Basis for Experts’ Judgments on the Consultation Content**

| **Basis for judgment** | **Degree (self-assessed)** | | |
| --- | --- | --- | --- |
|  | **High** | **Moderate** | **Low** |
| Practical or research experience |  |  |  |
| Theoretical knowledge and analysis |  |  |  |
| Relevant literature |  |  |  |
| Intuition |  |  |  |

**Table 6. Experts’ Familiarity with the Consultation Content**

| **Familiarity** | **Very familiar** | **Familiar** | **Moderately familiar** | **Slightly familiar** | **Unfamiliar** |
| --- | --- | --- | --- | --- | --- |
| Self-assessment |  |  |  |  |  |

**We welcome any additional comments or suggestions regarding this study:**

(This concludes the current round of expert consultation. Thank you for your valuable guidance and support.)
