# Supplementary material for: Development of a competency framework for public health nurses in China: A modified Delphi study
Source: PLoS One. 2026 Oct 30;21(10):e0361940. doi: 10.1371/journal.pone.0361940 (PMC13722293; doi:10.1371/journal.pone.0361940)
Supplement: Supplementary file 9 [file pone.0361940.s008.docx]

**S6 File. Expert Consultation Questionnaire for the Development of a Public Health Nursing**

**Competency Framework (Round 2)**

[English Translation]

Dear Expert,

Thank you for your continued participation in our study to develop a competency framework for public health nursing. We sincerely appreciate your valuable comments and guidance during the first round of expert consultation.

Following the first round, the competency framework was further revised based on expert feedback, discussion within the research team, and a review of relevant literature. Six competencies were removed; one domain, five subdomains, and two competencies were revised; four new competencies were added; three sets of competencies were merged; and the wording of several definitions was refined accordingly. In this round, we kindly invite you to evaluate the revised framework. Your comments and suggestions will provide an important basis for further refining the Public Health Nursing Competency Framework.

To facilitate the progress of the study, we kindly ask you to complete and return this round of the questionnaire within one week. If you have any questions or require further information, please feel free to contact us by telephone or email using the contact details provided at the end of the questionnaire. Thank you again for your valuable time and contribution. We sincerely look forward to receiving your response.

Sincerely,

XXX

Tel: XXXX

E-mail: XXX

**Instructions**

1. Tables 1–3 present the revised domains, subdomains, and competencies of the Public Health Nursing Competency Framework, with operational definitions provided for the competencies. We invite your comments on the inclusion, wording, and importance of each item.

2. The importance of each item is rated using a 5-point Likert scale: 5 = very important, 4 = important, 3 = moderately important, 2 = slightly important, and 1 = not important. Please rate the importance of each item by placing a “√” in the corresponding column or entering the corresponding score. Please select only one response for each item.

3. If you consider the wording of an item inaccurate or believe that an item should be removed, please provide your suggested revision or deletion in the “Comments and suggestions” column. If you believe that any important items have been omitted, please add them in the “Additional items” section and rate their importance using the same scale. Please provide a response for every item.

**Table 1. Expert Consultation on the Domains of the Public Health Nursing Competency Framework**

| **Domains** | **Importance rating** | | | | | **Comments and suggestions** |
| --- | --- | --- | --- | --- | --- | --- |
|  | **5** | **4** | **3** | **2** | **1** |  |
| **1. Community health surveillance and assessment** |  |  |  |  |  |  |
| **2. Health education and health promotion** |  |  |  |  |  |  |
| **3. Health management** |  |  |  |  |  |  |
| **4. Community public health emergency management** |  |  |  |  |  |  |
| **5. Partnership development and maintenance** |  |  |  |  |  |  |
| **6. Professionalism and professional development** |  |  |  |  |  |  |
| **Additional items** |  |  |  |  |  |  |

**Table 2. Expert Consultation on the Subdomains of the Public Health Nursing Competency Framework**

| **Domains** | **Subdomains** | **Importance rating** | | | | | **Comments and suggestions** |
| --- | --- | --- | --- | --- | --- | --- | --- |
|  |  | **5** | **4** | **3** | **2** | **1** |  |
| **1. Community health surveillance and assessment** | **1.1 Community health surveillance** |  |  |  |  |  |  |
|  | **1.2 Community health assessment** |  |  |  |  |  |  |
| **Additional items** |  |  |  |  |  |  |  |
| **2. Health education and health promotion** | **2.1 Health education** |  |  |  |  |  |  |
|  | **2.2 Health promotion** |  |  |  |  |  |  |
| **Additional items** |  |  |  |  |  |  |  |
| **3. Health management** | **3.1 Health information technology** |  |  |  |  |  |  |
|  | **3.2 Population-focused health management** |  |  |  |  |  |  |
|  | **3.3 Condition-specific health management** |  |  |  |  |  |  |
|  | **3.4 Setting-specific health management** |  |  |  |  |  |  |
|  | **3.5 TCM health management** |  |  |  |  |  |  |
| **Additional items** |  |  |  |  |  |  |  |
| **4. Community public health emergency management** | **4.1 Risk management** |  |  |  |  |  |  |
|  | **4.2 Emergency response** |  |  |  |  |  |  |
|  | **4.3 Recovery-phase management** |  |  |  |  |  |  |
| **Additional items** |  |  |  |  |  |  |  |
| **5. Partnership development and maintenance** | **5.1 Teamwork** |  |  |  |  |  |  |
|  | **5.2 Communication and coordination** |  |  |  |  |  |  |
| **Additional items** |  |  |  |  |  |  |  |
| **6. Professionalism and professional development** | **6.1 Professionalism** |  |  |  |  |  |  |
|  | **6.2 Professional development** |  |  |  |  |  |  |
| **Additional items** |  |  |  |  |  |  |  |

**Table 3. Expert Consultation on the Competencies of the Public Health Nursing Competency Framework**

| **Domains** | **Subdomains** | **Competencies and operational definitions** | **Importance rating** | | | | | **Comments and suggestions** |
| --- | --- | --- | --- | --- | --- | --- | --- | --- |
|  |  |  | **5** | **4** | **3** | **2** | **1** |  |
| **1. Community health surveillance and assessment** | **1.1 Community health surveillance** | **1.1.1 Knowledge of health surveillance:** Knowledge of core concepts and terminology in epidemiology, including commonly used measures of disease frequency, intensity and distribution. |  |  |  |  |  |  |
|  |  | **1.1.2 Ability to collect and manage health surveillance data:** The ability to systematically and continuously collect and manage health surveillance data on infectious diseases in the community (e.g., COVID-19, tuberculosis, HIV/AIDS, syphilis, hepatitis B), prevalent NCDs (e.g., type 2 diabetes, hypertension, malignant tumors), and community health hazards (e.g., food and drinking water safety risks, common school health problems and related risk factors, and reproductive health hazards). |  |  |  |  |  |  |
|  |  | **1.1.3 Ability to report and communicate health surveillance information:** The ability to compile and report surveillance data to public health authorities and communicate essential findings to community residents. |  |  |  |  |  |  |
|  | **Additional items** |  |  |  |  |  |  |  |
|  | **1.2 Community health assessment** | **1.2.1 Ability to conduct comprehensive community nursing assessments:** The ability to conduct systematic community health assessments by collecting and integrating multi-source data on community demographics, environmental conditions, social systems, and population health status and needs. |  |  |  |  |  |  |
|  |  | **1.2.2 Ability to analyze health data:** The ability to interpret and compare data obtained from community nursing assessments to analyze community health problems and health needs. |  |  |  |  |  |  |
|  |  | **1.2.3 Ability to develop community nursing diagnoses:** The ability to synthesize data from comprehensive community assessments to develop community nursing diagnoses, identify priority health problems, and guide the planning of public health nursing interventions. |  |  |  |  |  |  |
|  | **Additional items** |  |  |  |  |  |  |  |
| **2. Health education and health promotion** | **2.1 Health education** | **2.1.1 Ability to design health education programs:** The ability to design targeted health education programs based on community assessment data and informed by behavior change theories. |  |  |  |  |  |  |
|  |  | **2.1.2 Ability to develop health education materials:** The ability to develop accessible health education materials that are appropriate and tailored to the audience’s literacy level, language, culture, and cognitive capacity. |  |  |  |  |  |  |
|  |  | **2.1.3 Ability to implement health education programs:** The ability to use a range of health education methods (e.g., educational materials, public health consultations, group health talks, and individual counseling) to deliver targeted community health education. |  |  |  |  |  |  |
|  |  | **2.1.4 Ability to evaluate health education programs:** The ability to evaluate health education and health promotion programs across multiple dimensions, including reach, acceptability, effectiveness, efficiency, and equity. |  |  |  |  |  |  |
|  | **Additional items** |  |  |  |  |  |  |  |
|  | **2.2 Health promotion** | **2.2.1 Ability to engage in health advocacy:** The ability to raise community residents’ awareness of health issues through health advocacy activities and promote the development of a health-oriented community culture. |  |  |  |  |  |  |
|  |  | **2.2.2 Ability to conduct community mobilization:** The ability to motivate individuals, families, and the community to take action based on the health needs of community residents and work together toward shared community health goals. |  |  |  |  |  |  |
|  | **Additional items** |  |  |  |  |  |  |  |
| **3. Health management** | **3.1 Health information technology** | **3.1.1 Ability to use health information systems:** The ability to use public health information systems to establish, maintain, and standardize residents’ health records. |  |  |  |  |  |  |
|  |  | **3.1.2 Ability to deliver internet-enabled health services:** The ability to use internet-enabled platforms to remotely collect health information, conduct health assessments, and deliver personalized health guidance. |  |  |  |  |  |  |
|  | **Additional items** |  |  |  |  |  |  |  |
|  | **3.2 Population-focused health management** | **3.2.1 Ability to manage the health of children aged 0-6 years:** The ability to deliver comprehensive health management services for children aged 0-6 years, including newborn home visits, routine health checkups, immunization, and caregiver counseling on growth and development, nutrition, physical activity, and disease and injury prevention. |  |  |  |  |  |  |
|  |  | **3.2.2 Ability to manage women’s health:** The ability to assess women’s health status across key life stages (e.g., premarital, perinatal, and perimenopausal periods) and provide tailored guidance on lifestyle, sexual and reproductive health, and mental well-being. |  |  |  |  |  |  |
|  |  | **3.2.3 Ability to manage older adults’ health:** The ability to assess older adults’ lifestyle factors and health status and provide guidance on nutrition, physical activity, falls and injury prevention, immunization, medication safety, and mental health support. |  |  |  |  |  |  |
|  | **Additional items** |  |  |  |  |  |  |  |
|  | **3.3 Condition-specific health management** | **3.3.1 Ability to manage the health of people with NCDs:** The ability to conduct regular follow-up and routine monitoring, deliver nursing interventions, and provide self-management support for people with common chronic NCDs (e.g., hypertension and type 2 diabetes) to control risk factors and prevent complications. |  |  |  |  |  |  |
|  |  | **3.3.2 Ability to manage the health of people with infectious diseases:** The ability to provide referral, home visits (as appropriate), treatment adherence support, and follow-up for people with infectious diseases (e.g., tuberculosis and HIV), and deliver health education on prevention and control. |  |  |  |  |  |  |
|  |  | **3.3.3 Ability to manage the health of people with mental disorders:** The ability to manage case records, provide health monitoring for people with severe mental disorders, and deliver support for daily living skills and psychosocial rehabilitation. |  |  |  |  |  |  |
|  | **Additional items** |  |  |  |  |  |  |  |
|  | **3.4 Setting-specific health management** | **3.4.1 Ability to manage health in school settings:** The ability to collect and manage students’ health information, conduct systematic health assessments, and provide guidance and health education on growth and development, nutrition, physical activity, mental health, and sexual health. |  |  |  |  |  |  |
|  |  | **3.4.2 Ability to manage health in workplace settings:** The ability to contribute to occupational health surveillance and provide health education and counseling to workers on self-care, hazard identification, and injury prevention. |  |  |  |  |  |  |
|  | **Additional items** |  |  |  |  |  |  |  |
|  | **3.5 TCM health management** | **3.5.1 Knowledge of TCM health management:** Knowledge of core TCM health management theories, including Yin-Yang Theory, Five-Element Theory, Holism, Syndrome Differentiation, and Constitution Theory. |  |  |  |  |  |  |
|  |  | **3.5.2 Ability to provide TCM-based health guidance:** The ability to provide TCM health care guidance tailored to different body constitutions, including dietary regulation, daily routine adjustment, physical activity recommendations, and emotional regulation, and offer basic instruction on commonly used meridians and acupoints. |  |  |  |  |  |  |
|  | **Additional items** |  |  |  |  |  |  |  |
| **4. Community public health emergency management** | **4.1 Risk management** | **4.1.1 Ability to conduct community risk assessments:** The ability to contribute to community risk assessments for infectious disease outbreaks and public health emergencies, including assessments of environmental hazards, population exposures, and community preparedness and response capacity. |  |  |  |  |  |  |
|  |  | **4.1.2 Ability to develop emergency response plans:** The ability to contribute to the development, review, and updating of emergency response plans for infectious diseases and public health emergencies. |  |  |  |  |  |  |
|  |  | **4.1.3 Ability to provide community education on emergency management:** The ability to deliver community education to residents on emergency preparedness, emergency response, and self-help and mutual aid skills. |  |  |  |  |  |  |
|  | **Additional items** |  |  |  |  |  |  |  |
|  | **4.2 Emergency response** | **4.2.1 Ability to report emergency information:** The ability to report infectious disease events and public health emergencies accurately and promptly, in accordance with prescribed timelines, channels, and procedures. |  |  |  |  |  |  |
|  |  | **4.2.2 Ability to implement public health emergency response measures and deliver emergency medical care:** The ability to contribute to implementing emergency response measures (e.g., isolation, medical observation, emergency vaccination, and chemoprophylaxis when indicated), and providing emergency medical care (e.g., pre-screening, triage, first aid, and referral). |  |  |  |  |  |  |
|  |  | **4.2.3 Ability to conduct epidemiological investigations:** The ability to contribute to community-based field epidemiologic investigations, including case investigation, contact tracing, and data collection. |  |  |  |  |  |  |
|  |  | **4.2.4 Ability to collect clinical specimens:** The ability to contribute to the safe collection, labeling, storage, and transport of clinical specimens in accordance with standard protocols. |  |  |  |  |  |  |
|  |  | **4.2.5 Ability to support containment and environmental decontamination measures in outbreak-affected areas:** The ability to contribute to the implementation of containment measures and environmental cleaning and disinfection in outbreak-affected areas. |  |  |  |  |  |  |
|  |  | **4.2.6 Ability to provide psychological first aid:** The ability to provide psychological first aid and brief psychosocial support to affected individuals and families during and after public health emergencies, and provide referrals when necessary. |  |  |  |  |  |  |
|  | **Additional items** |  |  |  |  |  |  |  |
|  | **4.3 Recovery-phase management** | **4.3.1 Ability to conduct post-event evaluation:** The ability to conduct post-event evaluation of responses to infectious diseases and public health emergencies, to synthesize lessons learned, and to further improve emergency response plans. |  |  |  |  |  |  |
|  |  | **4.3.2 Ability to manage recovery-phase health issues:** The ability to manage the health issues of priority populations during the recovery phase of a public health emergency, addressing both physical rehabilitation needs and psychological recovery support. |  |  |  |  |  |  |
|  | **Additional items** |  |  |  |  |  |  |  |
| **5. Partnership development and maintenance** | **5.1 Teamwork** | **5.1.1 Ability to establish and sustain collaboration with external community partners:** The ability to proactively identify, engage, and sustain partnerships with external stakeholders (e.g., government and health authorities, public health agencies, and other healthcare organizations) to secure support and resources for community public health initiatives. |  |  |  |  |  |  |
|  |  | **5.1.2 Ability to collaborate within the community health team:** The ability to establish and sustain effective interprofessional collaboration within the community health team. |  |  |  |  |  |  |
|  |  | **5.1.3 Ability to collaborate with community residents:** The ability to encourage volunteers and community organizations to participate in community public health activities and provide relevant training for them. |  |  |  |  |  |  |
|  | **Additional items** |  |  |  |  |  |  |  |
|  | **5.2 Communication and coordination** | **5.2.1 Ability to conduct effective communication:** The ability to communicate effectively by applying active listening and providing clear, timely, and empathetic responses to facilitate mutual understanding and build rapport. |  |  |  |  |  |  |
|  |  | **5.2.2 Ability to align stakeholders:** The ability to align and coordinate multisectoral stakeholders using team-building, negotiation, conflict management, and facilitation skills. |  |  |  |  |  |  |
|  | **Additional items** |  |  |  |  |  |  |  |
| **6. Professionalism and professional development** | **6.1 Professionalism** | **6.1.1 Professional responsibility:** The ability to demonstrate commitment to public health nursing and a strong sense of professional responsibility. |  |  |  |  |  |  |
|  |  | **6.1.2 Legal literacy:** The ability to apply relevant laws, regulations, and policies to ensure compliance in public health nursing practice. |  |  |  |  |  |  |
|  |  | **6.1.3 Ethical literacy:** The ability to apply ethical principles (e.g., utility, respect, and health equity) to address ethical issues in public health nursing practice. |  |  |  |  |  |  |
|  |  | **6.1.4 Humanistic literacy:** The ability to integrate humanistic care into public health nursing practice and understand, appreciate, and respect cultural diversity. |  |  |  |  |  |  |
|  |  |  |  |  |  |  |  |  |
|  | **Additional items** |  |  |  |  |  |  |  |
|  | **6.2 Professional development** | **6.2.1 Ability to engage in self-directed learning:** The ability to engage in self-directed learning to maintain and enhance public health nursing knowledge and skills through multiple channels, including books, scholarly literature, online resources, academic exchanges, and continuing education. |  |  |  |  |  |  |
|  |  | **6.2.2 Ability to teach and mentor others:** The ability to educate and mentor students and novice nurses using appropriate pedagogical approaches. |  |  |  |  |  |  |
|  |  | **6.2.3 Ability to conduct continuous quality improvement:** The ability to conduct continuous quality improvement through a systematic process of data-driven gap identification, implementation of improvement strategies, and outcome monitoring. |  |  |  |  |  |  |
|  |  | **6.2.4 Ability to contribute to public health research projects:** The ability to participate in community public health research projects and to write and publish research papers in the field. |  |  |  |  |  |  |
|  | **Additional items** |  |  |  |  |  |  |  |

**We welcome any additional comments or suggestions regarding this study:**

(This concludes the current round of expert consultation. Thank you for your valuable guidance and support.)
