# Supplementary material for: Development of a competency framework for public health nurses in China: A modified Delphi study
Source: PLoS One. 2026 Oct 30;21(10):e0361940. doi: 10.1371/journal.pone.0361940 (PMC13722293; doi:10.1371/journal.pone.0361940)
Supplement: Supplementary file 11 [file pone.0361940.s010.docx]

# S3 Table. Mapping of competencies in the public health nursing competency framework to Bloom’s revised taxonomy

| **Domain** | **Subdomain** | **Competencies** | **Bloom’s revised taxonomy level** |
| --- | --- | --- | --- |
| 1. Community health surveillance and assessment | 1.1 Community health surveillance | 1.1.1 Knowledge of health surveillance | Remembering |
|  |  | 1.1.2 Ability to collect and manage health surveillance data | Applying |
|  |  | 1.1.3 Ability to report and communicate health surveillance information | Applying |
|  | 1.2 Community health assessment | 1.2.1 Ability to conduct comprehensive community nursing assessments | Analyzing |
|  |  | 1.2.2 Ability to develop community nursing diagnoses | Creating |
| 2. Health education and health promotion | 2.1 Health education | 2.1.1 Ability to design health education programs | Creating |
|  |  | 2.1.2 Ability to develop health education materials | Creating |
|  |  | 2.1.3 Ability to implement health education programs | Applying |
|  |  | 2.1.4 Ability to evaluate health education programs | Evaluating |
|  | 2.2 Health promotion | 2.2.1 Ability to engage in health advocacy | Applying |
|  |  | 2.2.2 Ability to conduct community mobilization | Applying |
| 3. Health management | 3.1 Health information technology | 3.1.1 Ability to use health information systems | Applying |
|  |  | 3.1.2 Ability to deliver internet-enabled health services | Applying |
|  | 3.2 Population-focused health management | 3.2.1 Ability to manage the health of children aged 0–6 years | Applying |
|  |  | 3.2.2 Ability to manage women’s health | Applying |
|  |  | 3.2.3 Ability to manage older adults’ health | Applying |
|  | 3.3 Condition-specific health management | 3.3.1 Ability to manage the health of people with NCDs | Applying |
|  |  | 3.3.2 Ability to manage the health of people with infectious diseases | Applying |
|  |  | 3.3.3 Ability to manage the health of people with mental disorders | Applying |
|  | 3.4 Setting-specific health management | 3.4.1 Ability to manage health in school settings | Applying |
|  |  | 3.4.2 Ability to manage health in workplace settings | Applying |
|  | 3.5 TCM health management | 3.5.1 Knowledge of TCM health management | Remembering |
|  |  | 3.5.2 Ability to provide TCM-based health guidance | Applying |
| 4. Community public health emergency management | 4.1 Risk management | 4.1.1 Ability to conduct community risk assessments | Analyzing |
|  |  | 4.1.2 Ability to develop emergency response plans | Creating |
|  |  | 4.1.3 Ability to provide community education on emergency management | Applying |
|  | 4.2 Emergency response | 4.2.1 Ability to report emergency information | Applying |
|  |  | 4.2.2 Ability to implement public health emergency response measures and deliver emergency medical care | Applying |
|  |  | 4.2.3 Ability to conduct epidemiological investigations | Applying |
|  |  | 4.2.4 Ability to collect clinical specimens | Applying |
|  |  | 4.2.5 Ability to support containment and environmental decontamination measures in outbreak-affected areas | Applying |
|  |  | 4.2.6 Ability to provide psychological first aid | Applying |
|  | 4.3 Recovery-phase management | 4.3.1 Ability to conduct post-event evaluation | Evaluating |
|  |  | 4.3.2 Ability to manage recovery-phase health issues | Applying |
| 5. Partnership development and maintenance | 5.1 Teamwork | 5.1.1 Ability to establish and sustain collaboration with external community partners | Applying |
|  |  | 5.1.2 Ability to collaborate within the community health team | Applying |
|  | 5.2 Communication and coordination | 5.2.1 Ability to conduct effective communication | Applying |
|  |  | 5.2.2 Ability to align stakeholders | Applying |
| 6. Professionalism and professional development | 6.1 Professionalism | 6.1.1 Professional responsibility | Understanding |
|  |  | 6.1.2 Legal literacy | Understanding |
|  |  | 6.1.3 Ethical literacy | Understanding |
|  |  | 6.1.4 Humanistic literacy | Understanding |
|  | 6.2 Professional development | 6.2.1 Ability to engage in self-directed learning | Applying |
|  |  | 6.2.2 Ability to teach and mentor others | Applying |
|  |  | 6.2.3 Ability to conduct continuous quality improvement | Creating |
|  |  | 6.2.4 Ability to contribute to public health research projects | Creating |
