# Supplementary material for: Development of a competency framework for public health nurses in China: A modified Delphi study
Source: PLoS One. 2026 Oct 30;21(10):e0361940. doi: 10.1371/journal.pone.0361940 (PMC13722293; doi:10.1371/journal.pone.0361940)
Supplement: Supplementary file 14 [file pone.0361940.s014.doc]

The authors have responded thoroughly to the previous comments. The manuscript is much improved, and most major concerns have been addressed. Some points still need attention.

**1. Definition of public health nurse**

The Introduction now states that public health nurse is not an official professional title in China and that the term refers to nurses in primary health care institutions who carry out public health nursing duties. The main responsibilities are also listed. This largely addresses my earlier concern. I suggest one additional sentence to clarify how this term relates to actual job titles in China, for example that such staff may be called community nurses, public health nursing post holders, or similar titles. This would help readers understand that public health nurse is an operational term rather than a formal title.

**2. Deepening the Discussion**

The response letter explains that international frameworks emphasize broad professional competencies, while the Chinese framework emphasizes frontline service delivery under the National BPHS, and that TCM health management is part of the BPHS. This explanation is still brief and does not fully explain why some competencies are emphasized and others receive lower weights. I suggest adding a subsection or several paragraphs on international comparison and policy and role differences, linking the weights to the policy system and the role boundaries of PHNs. For example:

(1) In this study, health education and health promotion received the highest weight (0.280); health management and community health surveillance and assessment both received 0.196; community public health emergency management, partnership development and maintenance, and professionalism and professional development each received 0.110. TCM health management received a relatively low subdomain weight of 0.081 and a global weight of 0.016; the subdomain mean was 4.48, and the means for its two competencies were 4.43 and 4.52. Note that 4.38 belongs to 3.4.2 Ability to manage health in workplace settings, not to TCM health management.

(2) International frameworks, such as those of the United States, Canada, and Japan, often emphasize policy and program planning, financial management, systems leadership, and communication and collaboration. In the Chinese framework, policy, financial management, and systems leadership are relatively weak or do not appear as separate competencies. This may reflect that PHNs in China currently focus mainly on implementing the standardized National BPHS rather than taking part in policy making, financial management, and systems leadership.

(3) Although TCM health management is part of the National BPHS, the Delphi experts gave it a relatively low subdomain weight. This may indicate that PHNs have limited autonomy in TCM health management and mainly assist, refer, or provide health education rather than independently providing core TCM diagnosis and treatment. The authors should explain this explicitly in the Discussion. Otherwise, readers may ask why TCM health management is assigned a relatively low subdomain weight despite being a BPHS item.

**3. Inconsistent terminology**

The revised manuscript still uses inconsistent terminology. Reviewer #2 asked for consistent use of domains, subdomains, and competencies, but this has not been fully implemented. For example, the Abstract contains the following formulations:

(1) six domains, 16 subdomains, and 52 competencies

(2) six primary domains, 16 secondary indicators, and 52 tertiary indicators

(3) six primary, 16 secondary, and 46 tertiary indicators

**Recommendation: minor revision.**
