# Supplementary material for: Development of a competency framework for public health nurses in China: A modified Delphi study
Source: PLoS One. 2026 Oct 30;21(10):e0361940. doi: 10.1371/journal.pone.0361940 (PMC13722293; doi:10.1371/journal.pone.0361940)
Supplement: Supplementary file 12 [file pone.0361940.s015.docx]

**Response to Reviewer #1:**

**MAJOR COMMENTS**

1. the competency framework is structured into three levels (domains, sub-domains, and tertiary indicators). The third-level indicators are detailed in the Supplementary Materials. However, a major area for methodological improvement is that these third-level descriptions are predominantly descriptive and task-oriented, lacking a rigorous taxonomic structure. I fully recognize that the current wording is the direct outcome of the Delphi consensus. Consequently, the authors cannot arbitrarily rewrite these items using taxonomic verbs without invalidating the study results. To reconcile the finalized Delphi consensus with the need for educational and taxonomic rigor, I suggest addressing this limitation through one or both of the following actions:

(a) add supplementary columns to the framework that retroactively classify each item by its conceptual domain and map it to a recognized taxonomic framework (e.g., Bloom's taxonomy) to act as an interpretative layer without altering the original text and the study findings;

(b) explicitly address this constraint in the Limitations section, underlining that translating these functional tasks into pedagogically measurable competencies, with clearly defined levels of nursing autonomy, will be a fundamental and necessary step for future research.

**Response:**

Thank you for this valuable comment. We agree that the Delphi-derived competencies primarily represent functional competency statements and that their wording should not be arbitrarily modified after consensus was reached.

To address this concern, we added **S3Table, “Mapping of competencies in the public health nursing competency framework to Bloom’s revised taxonomy.”** The competencies were mapped to Bloom’s revised taxonomy as an interpretative layer without modifying the original Delphi-derived statements. The corresponding description was added in the Results section **(Lines 525-527).**

In addition, we have revised the Limitations section to acknowledge that the competencies need to be further translated into measurable learning outcomes **(Lines 768-770).**

**MINOR COMMENTS**

2. the first opening paragraph of the Introduction seems a bit broad and not perfectly aligned with the study's objectives. While citing these global issues creates a sense of urgency, these concepts are not well integrated with the rest of the text. I suggest rewriting this first paragraph to be more brief.

**Response:** Thank you for this valuable comment. We revised the opening section of the Introduction to improve its focus and alignment with the study objectives. The global background was condensed to retain only information directly relevant to public health workforce development. The revised text can be found in **Lines 62-72.**

3. on page 8, the relation between community nursing and public health nursing is introduced, however a specific supporting reference is missing;

**Response:**Thank you for this helpful comment. We have added representative references to support the two conceptual models describing the relationship between community nursing and public health nursing.The revised text and the added references can be found in **Lines 217-223.**

4. in the Methods section, the authors mention the use of "maximum variation purposive sampling". I suggest adding a brief rationale and a supporting methodological reference;

**Response:**Thank you for this helpful comment. We have added a brief rationale for using maximum-variation purposive sampling and included a methodological reference to support this sampling strategy. The revised text and added reference can be found in **Lines 298-302.**

5. in the demographic tables, mirroring data (e.g., male/female) is redundant. I suggest collapsing these variables to improve readability;

**Response:**Thank you for this helpful suggestion. We carefully considered this recommendation. As only the gender variable consisted of two mutually exclusive categories, whereas all other demographic variables included three or more categories, we retained the current table format to ensure consistency and facilitate comparison across variables.

6. according to what was introduced in the background, the division of the practitioners' field of expertise into "community nursing", "public health", and "health education" appears unclear. Given that these fields heavily overlap (as noted in the Background), I suggest reformulating and restructuring this categorization to avoid taxonomic ambiguity or providing a rationale;

**Response:** Thank you for this insightful comment. We agree that the original categorization of the experts' field of expertise could lead to taxonomic ambiguity. Therefore, we revisited the original data and reclassified the experts according to their primary professional field, resulting in four mutually exclusive categories: community nursing, public health, primary care medicine, and nursing education & related specialties. Specifically, community nursing includes experts engaged in frontline community nursing practice, chronic disease management, geriatric nursing, and community nursing management; public health includes experts from Centers for Disease Control and Prevention and those specializing in public health, health policy, and social medicine; primary care medicine includes experts in general practice/family medicine; and nursing education & related specialties includes experts in nursing education, nursing management, psychiatry, and related specialties. The revised table and corresponding text can be found in **Lines 466-470.**

7. the text cites the high response rate as evidence of the Delphi process's quality. However, with a purposively selected sample of 22 subjects, the response rate should not be presented as the primary indicator of strength. I suggest shifting the emphasis away from the sample size and instead highlighting the high quality, expertise, and specific competencies of the professionals involved in the panel.

**Response:** Thank you for this valuable comment. We revised the Discussion to emphasize the qualifications and multidisciplinary composition of the Delphi panel, while presenting the response rate as supporting evidence for the Delphi consultation process rather than its primary strength. In addition, the Statistical Analysis section was revised to describe expert engagement, the expert authority coefficient, and the coordination of expert opinions as characteristics of the Delphi consultation process, rather than indicators used to assess its quality. The revised text can be found in **Lines 469-581 and Lines 372-375 .**

**Response to Reviewer #3:**

1. Clearly define the concept of "public health nurse"

The term "public health nurse" is not commonly used in China's healthcare setting. Although the authors mention the relationship between public health nursing and community nursing in the introduction, this relationship is still not very clear to readers. I suggest that the authors provide a clearer definition of "public health nurse" in the introduction section, explaining how this role is understood and positioned within China's current health system.

**Response:** Thank you for this valuable comment. We revised the Introduction to provide a clearer operational definition of public health nurse (PHN) in the context of China's healthcare system. Specifically, we clarified that public health nurse is not an officially designated professional title in China but is used in this study to refer to nurses working in primary health care institutions who undertake public health nursing responsibilities. We also described the scope of their primary responsibilities to clarify how this role is understood and positioned within China's current healthcare system. The revised text can be found in **Lines 81-87.**

2. Limitations regarding the representativeness of the expert panel

2.1. Geographic imbalance: Among the 21 experts, 52.4% came from Western China. Given the notable differences in healthcare development levels between Eastern and Western China, the results may be biased toward experiences from less developed regions, and the framework's applicability to more developed Eastern regions remains uncertain. The authors should mention this in the limitations section.

**Response:** Thank you for this valuable comment. Following your suggestion, we revised the Limitations to acknowledge the potential limitation of the geographic distribution of the Delphi panel. Although experts from four major economic regions of China were included, more than half were from Western China, future research should further validate and refine the framework through broader regional representation. The revised text can be found in **Lines 763-768 and Lines 771-775.**

2.2. Professional background imbalance: Most of the authors' experts (57.1%) had a background in community nursing, while only 14.3% had a background in public health. The competency framework the authors developed is for public health nurses, so would it not be more appropriate to have a stronger public health perspective rather than a nursing perspective? I suggest that the authors acknowledge this bias in the limitations and note that future studies should include more experts from public health, health management, epidemiology, and other related fields.

**Response:** Thank you for this valuable comment. Following your suggestion, we re-examined the professional backgrounds of all Delphi experts according to their primary professional field and revised Table 1 accordingly. The updated classification includes experts from community nursing (47.6%), public health (23.8%), primary care medicine (9.5%), and nursing education and related specialties (19.0%), providing a broader multidisciplinary representation than the original classification. Nevertheless, we acknowledge that community nursing remained the largest disciplinary group within the Delphi panel. We have therefore added this as a limitation and noted that future research should further strengthen multidisciplinary representation by including experts from additional public health–related disciplines. The revised text can be found in **Lines 763-768 and Table 1.**

3. Deepen the discussion section

The authors compared their framework with those from the United States, Canada, and Japan, but the comparison mainly stays at the level of "similarities". I think the authors need to go deeper into why certain competencies are emphasized in China (such as 3.5 TCM health management competency) while others are given less weight. A discussion of how differences in policy systems or nursing role definitions across countries might explain these differences would strengthen this section.

**Response:**

Thank you for this valuable comment. Following your suggestion, we revised the Discussion to clarify that international competency frameworks generally emphasize broad professional competencies across diverse public health nursing roles and practice settings. In contrast, the framework developed in this study places particular emphasis on competencies relevant to frontline service delivery under China’s National Basic Public Health Services (BPHS), reflecting the current role of PHNs in implementing standardized public health services within primary health care settings. The revised text can be found in **Lines 719-736.**

Regarding the TCM health management competency raised in your comment, it is an established component of the BPHS, and its inclusion in the framework reflects the routine responsibilities of PHNs in China's primary health care system.

4. Conclusion needs clearer guidance for next steps

The conclusion states that the framework demonstrates methodological rigor and contextual relevance. I suggest a more cautious tone, clearly stating that this framework has not yet been empirically validated. The authors should specify what should be done next, for example: testing the reliability and validity of the framework, pilot applications in real settings, and developing tiered competency standards for different career levels.

**Response:** Thank you for this valuable comment. Following your suggestion, we revised the Conclusion to adopt a more cautious interpretation of the proposed competency framework. Specifically, we removed statements that could be interpreted as implying that the framework has already demonstrated methodological rigor or contextual applicability, and instead emphasized that it provides a structured reference for competency development. We also explicitly acknowledged that the framework has not yet been empirically validated in practice. In addition, we further clarified future research priorities, including broader regional and multidisciplinary representation, pilot implementation in practice settings, psychometric validation, and the development of measurable learning outcomes and tiered competency standards. The revised text can be found in **Lines 771-784.**

**Response to Reviewer #2:**

**Major issues/Major concerns**

**1. Introduction. Rationale asserted rather than substantiated.** The statement that “(…) because of differences in healthcare systems, sociocultural contexts, and stages of development, these frameworks cannot be directly applied in China" (lines 103–105) is presented as a given. As this assertion is central to the rationale for the study, it would benefit from substantiation rather than being stated as self-evident. The argument would be considerably strengthened were the authors to elaborate on this claim by identifying the specific differences, and similarities, between the international frameworks (e.g., those of the United States, Canada, and Japan) and the Chinese context - for instance, in the organization of primary care, the role boundaries between public health and community nursing, and the sociocultural expectations of the nursing role - and by explaining how each of these factors would bear upon the design and implementation of a competency framework for PHNs in China. Such an elaboration would render the case for a China-specific framework explicit rather than assumed.

**Response:** Thank you for this valuable suggestion. We have revised the Introduction to provide a clearer rationale for why existing international competency frameworks cannot be directly applied in China. Specifically, we added a comparison between representative international public health nursing practice models (i.e., the Minnesota Public Health Interventions Wheel in the United States and the concentric-circle practice methodology in Japan) and the current practice context in China. The revised text highlights that the content and structure of competency frameworks are fundamentally shaped by the scope and orientation of public health nursing practice within different healthcare systems. While international frameworks emphasize comprehensive practice extending from individuals and families to communities and health systems, public health nursing practice in China is primarily organized around the nationally standardized Basic Public Health Services (BPHS) programme delivered through primary health care institutions. We therefore clarified that, although international frameworks provide valuable conceptual guidance, differences in the scope and orientation of public health nursing practice necessitate contextual adaptation rather than direct transfer. These revisions have been incorporated into the Introduction **(Lines 144-165).**

**2. Cross-cutting issue. Reconsider the use of the term "indicators."** The framework is described as comprising "six primary domains, 16 secondary indicators, and 46 tertiary indicators." The term "indicators" may not best convey the constructs being described, which are competencies rather than measurement indicators. Consistent and accurate terminology is important for conceptual clarity and for alignment with the competency frameworks’ literature. The authors should reconsider the terminology, for example adopting a hierarchy such as domains → competencies → sub-competencies, and apply it consistently throughout the manuscript.

**Response:** Thank you for this insightful suggestion. We agree that the term *indicators* may be interpreted as referring to measurement indicators and does not accurately reflect the hierarchical structure of a competency framework. To improve conceptual clarity and align the terminology with the competency framework literature, we have revised the framework terminology throughout the manuscript. Specifically, the hierarchical structure has been changed from *primary, secondary, and tertiary indicators* to *domains, subdomains, and competencies*, respectively. Corresponding revisions have been made consistently throughout the text, tables, figures, and supplementary materials.

**3. Methods. Incorrect attribution of the definition of ‘competency’.** The definition provided, namely that "competency is defined as the systematic integration of knowledge, skills, judgment and personal attributes (including attitudes, values and beliefs) that professionals require to fulfil their role responsibilities and achieve desired practice outcomes" (lines 157–160), is attributed to McClelland, 1973. This attribution appears questionable: McClelland's seminal work introduced the general concept of testing for competence, whereas the wording adopted here corresponds far more closely to the competence definition formulated by the Canadian Nurses Association. Because an accurate conceptual foundation is essential to the validity of the framework, the authors should verify the source and cite the definition correctly.

**Response:** Thank you for identifying this important conceptual issue. We agree that the original citation of McClelland (1973) for the specific definition of competency was inappropriate. We have therefore revised the text by defining competency with reference to the ICN Framework of Competencies for the Nurse Specialist and updated the corresponding citation accordingly. The revised text can be found in **Lines 231-236.**

**4. Methods. Insufficient reporting of the literature review.** The search is described as "systematic," yet no structured search strategy, PRISMA flow diagram, screening protocol, number of reviewers, or procedure for resolving disagreements is reported. Furthermore, only a raw count is provided (9547 records reduced to 40 included), with no breakdown of records excluded at each stage. These omissions prevent readers from appraising the comprehensiveness and reproducibility of the evidence base underpinning the preliminary framework. The authors should report the full search strategy, present a PRISMA-style flow diagram documenting records at each stage, and describe the screening procedure, including the number of reviewers involved and the method for resolving disagreement.

**Response:** Thank you for this constructive suggestion. We have revised the manuscript to improve the transparency and reproducibility of the literature review process. Specifically, we have added the detailed search strategies for all databases in **S2 File** and a PRISMA-style flow diagram illustrating the study selection process as **S1 Fig**. In addition, the Methods section has been revised to describe the literature screening procedure, including independent screening by two reviewers and resolution of disagreements through discussion. The Results section has also been updated to report the numbers of records excluded at each stage of the screening process. The corresponding revisions can be found in **Lines 263-266 and Lines 408-417.**

**5. Methods. Specify the type of "job analysis" performed.** The procedure labelled "job analysis" (lines182–197) derives competency items from two national policy documents (the National Basic Public Health Service Standards, 3rd ed., and the Specifications for Newly Included Basic Public Health Service–Related Work). Because job analysis may be conducted through several distinct methods (e.g., document or standards review, observation, work sampling, or incumbent surveys), the lack of an explicit designation could lead readers to assume that a field-based analysis was undertaken. To aid transparency, the authors should state explicitly that a document-based job analysis was performed, briefly describe how the described job specification roles and tasks were extracted and translated into competency statements, and clarify how this step relates to the subsequent practitioner interviews.

**Response:** Thank you for this helpful suggestion. We have revised the Methods section to specify that a document-based job analysis was conducted using the two national policy documents. We have also clarified that the roles, responsibilities, and service tasks described in these documents were interpreted as the knowledge, skills, and abilities required for public health nursing practice and expressed as competency items.The corresponding revisions can be found in **Lines 276-280.**

Regarding the relationship between the document-based job analysis and the practitioner interviews, these were not sequential steps. Rather, together with the literature review, they served as three complementary sources of data for competency development. As described in the Results **(Lines 444-451)**, these data were collated using standardized extraction forms and subsequently integrated through directed content analysis to identify and classify competency indicators, resulting in the preliminary competency framework.

**6. Methods. Lack of an explicit qualitative research design.** The semi-structured interviews are described procedurally, but no underlying theoretical or methodological orientation (e.g., phenomenology, grounded theory, reflexive thematic analysis) is identified. As the chosen orientation shapes the design of the interview guide, the sampling logic, and the mode of analysis, its omission limits the reader's ability to judge the coherence of the qualitative component. The authors should state the theoretical grounding that informed the conduct and analysis of the interviews.

**Response:** Thank you for this valuable suggestion. We have revised the Methods section to clarify the methodological orientation of the qualitative component. Specifically, a qualitative descriptive approach was adopted to explore practitioners’ perspectives on public health nursing competency requirements, with reference to Sandelowski’s work on qualitative description. The interview data were subsequently analyzed using thematic analysis. The corresponding revisions can be found in **Lines 290-297.**

**7. Methods. COREQ checklist for the interviews.** A completed COREQ (Consolidated Criteria for Reporting Qualitative Research) checklist (Tong et al., 2007) does not appear to be included. Given that the interviews materially shaped the preliminary framework, adherence to an established qualitative reporting standard is important for transparency and appraisal. The authors should complete and provide a COREQ checklist as supporting information.

**Response:** Thank you for this constructive suggestion. We have completed the Consolidated Criteria for Reporting Qualitative Research (COREQ) checklist and provided it as **S4File**. In addition,we have added relevant information regarding the qualitative approach, interview procedures, and data analysis to the Methods section **(Lines 290-335).**

**8. Methods. Non-nurse perspectives in a nursing competency framework.** The interviewees comprised physicians and administrators in addition to nurses (6 physicians, 11 nurses, 5 administrators), meaning that a framework of nursing competencies is partly grounded in non-nurse perspectives. Without a conceptual justification, it is unclear how the input of non-nurse participants maps onto PHN-specific competencies, which raises a question of construct validity. The authors should justify the inclusion of these groups and explain how their contributions were interpreted and integrated in relation to public health nursing competencies.

**Response:** Thank you for this important comment. We would like to further clarify the rationale for including non-nurse participants in the qualitative component. Public health nursing practice in China is delivered within primary health care teams, where nurses work closely with physicians and administrative staff in the implementation, coordination, and management of population-based public health services. Therefore, we included community physicians, nurses, and nursing managers to capture complementary perspectives from frontline practice, interprofessional collaboration, and nursing management.

**9. Methods — Reporting and trustworthiness of the thematic analysis.** The thematic analysis is reported as having used NVivo 11, but no coding framework, number of coders, measure of inter-coder reliability, or audit trail is described, and trustworthiness criteria (e.g., credibility, confirmability, member checking) are not addressed. The absence of these elements undermines confidence in the rigour of the qualitative analysis. The authors should describe the coding process, the number and roles of coders, the procedures used to establish trustworthiness, and any audit trail maintained.

**Response:** Thank you for this important comment. We have further clarified the procedures used for thematic analysis and the measures taken to enhance the rigour of the qualitative analysis. Two researchers with qualitative research training independently reviewed and coded the interview transcripts using NVivo 11. Codes were iteratively categorized and refined into competency-related categories, with discrepancies resolved through discussion until consensus was reached.The corresponding clarification has been added to the Methods section **(Lines 328-335).**

We acknowledge that formal inter-coder reliability testing and an independent audit trail were not established during the interview data analysis. However, independent coding by two researchers, resolution of coding discrepancies through discussion, and the integration of interview findings with evidence from the literature review and document-based job analysis enhanced the methodological rigour of the preliminary framework development process.

**10. Methods. Potentially leading interview guide.** The four domains of the interview guide appear partly leading (e.g., "competency requirements… and their specific components"), which may have primed participants toward a pre-existing competency structure rather than eliciting open accounts. This risks circularity, whereby the interviews confirm rather than inform the framework. The authors should reflect on this possibility, and ideally provide the interview guide (see comment 10) so that the degree of openness can be assessed.

**Response:** Thank you for this important comment. We agree that the wording of the original description of the interview domains may have unintentionally implied that the interviews were designed to confirm a predefined competency structure. We have revised the description to better reflect the exploratory nature of the qualitative component **(Lines 311-319)**.

The purpose of the interviews was to explore practitioners’ experiences, perceptions, and expectations regarding public health nursing practice and competency requirements. Although the interview guide focused on competency-related topics in line with the study objective, no predefined competency domains or indicators were provided to participants during the interviews. The interview guide has been provided as **S3 File** to facilitate assessment of the openness of the interview process.

**11. Methods. Provide the interview guide.** The interview guide is referred to but not supplied. As the guide is central to appraising the qualitative component and the concern raised in comment 9, its inclusion as supporting information would substantially enhance transparency. The authors should provide the full interview guide.

**Response:** Thank you for this suggestion. The full semi-structured interview guide has now been provided as **S3 File.**

**12. Methods. Provide the completed CREDES checklist.** The manuscript states that the process followed the CREDES reporting checklist, yet a completed checklist does not appear to be included. Without it, readers cannot verify the extent of adherence to the standard. The authors should provide the completed CREDES checklist as supporting information.

**Response:** Thank you for this suggestion. The completed CREDES checklist has been provided as **S1 File**, and the corresponding statement has been added to the Methods section **(Lines 191-194).**

**13. Methods. Rationale for using AHP.** The manuscript applies AHP to derive indicator weights but does not explain why this method was selected in preference to other multi-attribute value measurement approaches. As the choice of weighting method influences the results and their interpretation, a justification is warranted. The authors should briefly explain what AHP is, state why it was chosen over alternative methods, and acknowledge its principal strengths and limitations.

**Response:** Thank you for this important comment. We have added a brief explanation of the Analytic Hierarchy Process (AHP) in the Methods section **(Lines 389-393)**. AHP was selected because it enables systematic comparison and weighting of hierarchical indicators based on expert judgments, which was consistent with the purpose of this study and the characteristics of the competency framework. Its major advantage is that it provides a structured weighting procedure and incorporates consistency testing to improve the reliability of the weighting results. Considering the overall coherence and scope of the manuscript, we did not add a separate discussion of AHP-specific limitations; however, the limitations related to expert-based judgment and framework validation have been addressed **(Lines 768-770).**

**14. Methods. Transparency of the AHP weighting procedure.** The AHP weighting is underspecified: the Methods state only that "AHP was used to determine the weights." Because the relative weights are a central deliverable of the study, this lack of detail is a significant limitation; it is unclear who performed the pairwise judgments, how many respondents contributed to each matrix, how individual judgments were aggregated, and at what point in the process the comparisons were collected. Since the validity and interpretability of the weights depend entirely on this procedure, its omission constrains appraisal of the results. The authors should describe the AHP elicitation in full, including the respondents and their number, the comparison scale used, the aggregation method, and the manner in which consistency (CR < 0.10) was handled for any matrices that initially failed.

**Response:** Thank you for this important comment. We agree that the previous description of the AHP weighting procedure was insufficiently detailed. We have revised the Methods section to provide further clarification of the AHP weighting procedure, including the source of expert judgments, the comparison scale, the aggregation process, and the consistency assessment **(Lines 389-400)**. All judgment matrices met the predefined consistency criterion (CR < 0.10), and no further adjustment was required. The characteristics and number of Delphi experts involved in the weighting process are reported in the Results section **(Lines 455-470).**

**15. Methods/Results. Low Kendall's coefficient of concordance versus the claim of strong consensus.**The reported Kendall's W values are 0.156 (Round 1) and 0.171 (Round 2). Although both are statistically significant, the significance here is largely a function of the number of items rather than the strength of agreement, and values in this range conventionally indicate only weak overall concordance among experts. This sits in tension with the manuscript's repeated framing of the process as demonstrating "strong expert consensus" and "increasing agreement across rounds," given that the increase from 0.156 to 0.171 is marginal and the absolute level remains low. The authors should (i) explicitly acknowledge and interpret the low W values, (ii) distinguish between item-level consensus (the mean and CV thresholds, which were met) and overall opinion coordination (W, which was weak), and (iii) discuss what the low W implies for the robustness of the framework. Reporting W alongside accepted interpretive benchmarks would substantially improve the credibility of this section.

**Response:** Thank you for this important comment. We agree that Kendall’s W reflects overall coordination among experts rather than item-level consensus, and that the previous wording may have overstated the strength of agreement. We have revised the Discussion to acknowledge the relatively low W values and clarify the distinction between overall opinion coordination and item-level consensus based on the predefined criteria of mean importance ratings and coefficients of variation **(Lines 572-581).**

**16. Methods. Representativeness of the expert panel.** The final panel (n = 21) is comparatively small for a framework intended to be nationally applicable and is notably skewed in both geography and discipline: 52.4% of experts were from Western China and only 9.5% from Central China, while 57.1% specialized in community nursing, with public health and general practice represented by only three experts each (14.3%). This imbalance could bias which competencies are prioritized and weighted, thereby limiting the national generalizability of the framework. The authors should explicitly acknowledge this distribution as a limitation.

**Response:** Thank you for this important comment. We have acknowledged the potential influence of the geographical and disciplinary distribution of the Delphi panel as a limitation and added this clarification in the Limitations section **(Lines 763-768).**

**17. Results. Output of the literature review.** The Results section do not report which competencies, or how many, emerged from the analysis of the retrieved studies. As the literature review is one of the three pillars of the preliminary framework, this omission leaves a gap. The authors should report the competencies derived from the literature review and their number.

**Response:** Thank you for this comment. We have added a supplementary table to provide further details on the literature review and improve the transparency of this component of the preliminary framework development.

We would also like to clarify that competency indicators were not generated separately from the literature review. As described in the Results **(Lines 444-451)**, data from the literature review and other sources were collated using standardized extraction forms and integrated for analysis. Guided by the theoretical frameworks, we then conducted directed content analysis to identify and classify competency indicators. Therefore, it is not possible to attribute a specific number of competency indicators exclusively to the literature review. The supplementary table has been added in **S1 Table**, and the relevant revisions can be found in **Lines 416-417**.

**18. Results. Present the preliminary framework.** The text states that the process "yielded a preliminary three-tier competency framework for public health nursing, comprising six primary, 16 secondary, and 52 tertiary indicators," but this preliminary framework is not presented. Without it, readers cannot trace how the framework evolved through the Delphi rounds. The authors should present the full preliminary three-tier framework (e.g., as a table/figure within the manuscript or as a supplementary material file).

**Response:** Thank you for this suggestion. The complete preliminary three-tier competency framework has now been provided as part of the first-round expert consultation questionnaire in **S5 File**. The revised text can be found in **Lines 447-451.**

**19. Results. Professional groups of the Delphi participants.** The Delphi results do not detail the professional groups of the expert panel beyond broad fields of expertise. As the credibility of a Delphi study depends heavily on panel composition, detailed reporting is warranted. The authors should describe the professional groups represented among the participants. For instance, it would be relevant to know how many nurses participated in the Delphi process.

**Response:** Thank you for this comment. We have added the professional groups of the Delphi experts to Table 1 and provided the corresponding information in the Results section. Among the 21 experts, 14 (66.7%) were nurses, three (14.3%) were physicians, and four (19.0%) were public health physicians. The revised text and Table 1 can be found in **Lines 466-470.**

**20. Results. Present the Delphi questionnaires across the several rounds.** The manuscript would benefit from presentation of the web-Delphi questionnaire screens used across the several rounds. Doing so would allow readers to understand how items were presented and rated and would enhance transparency. The authors could include representative questionnaire screens as supporting material.

**Response:** Thank you for this suggestion. To enhance the transparency of the Delphi process and show how the competency indicators were presented and rated, we have added the complete expert consultation questionnaires used in both Delphi rounds as supporting materials **(S5 and S6 Files).** A corresponding statement has also been added to the Results section. The revised text can be found in **Lines 447-451 and Lines 508-510.**

**22. Results. Rationale for items changes.** The statement that, "based on expert feedback and discussion within the research team, six tertiary indicators were removed" (and that others were revised, merged, or added) does not explain the basis for these decisions. Without explicit decision rules, the modifications may appear post-hoc and reduce procedural transparency. The authors should explain the criteria and reasoning underpinning the removal, addition, merging, and revision of indicators across rounds.

**Response:** Thank you for this comment. We have clarified the criteria and decision-making process underlying indicator modifications across the Delphi rounds. All indicators met the predefined quantitative criteria in both rounds. Indicator modifications were therefore guided primarily by experts’ qualitative feedback and research team discussion. Each expert suggestion was reviewed, with consideration of conceptual overlap, clarity and precision of wording, and consistency with the defined scope of public health nursing; relevant policy documents, literature, and findings from the preliminary fieldwork were also considered when applicable. We have added the rationale for the indicator changes across both rounds in the Results section. The revised text can be found in **Lines 496-508 and Lines 512-516.**

**23. Results. Define the pre-specified stopping criteria.** The sentence "The pre-specified stopping criteria were met; therefore, no further Delphi rounds were conducted" does not state, at that point, what those criteria were. Because the decision to halt the process bears directly on the validity of the claimed consensus, the criteria should be unambiguous. The authors should clearly state the pre-specified stopping criteria and confirm that they were defined a priori.

**Response:** Thank you for this comment. The stopping criteria were defined a priori and are specified in the Delphi procedures section of the Methods (**Lines 364-368)**. Specifically, the Delphi process was concluded when the mean importance rating for each item was ≥ 4.0, the CV was ≤ 0.25, and expert comments were largely limited to minor wording refinements. In the second round, all indicators met the quantitative criteria, with mean importance ratings ranging from 4.38 to 5.00 and CVs ranging from 0.00 to 0.18, while expert feedback was substantially reduced and focused primarily on further refinement of the framework. Therefore, the pre-specified stopping criteria were considered met, and no further Delphi rounds were conducted.

**24. Discussion. Contributions and implications.** The Discussion would benefit from a clearer articulation of the study's contributions and implications for both research and practice. In particular, it remains underdeveloped how the findings will be used to strengthen PHN workforce development in China. The authors should explain how the framework can inform education, training, assessment, and policy, and discuss the next steps required to operationalize it.

**Response:** Thank you for this comment. We have expanded the Discussion by adding a subsection entitled “Implications for PHN workforce development” to clarify the contributions and practical implications of the framework. The revised text can be found in **Lines 745-755.**

**25. Discussion. Strengthen the Discussion, section including the ‘Study Limitations’ sub-section.** The Discussion, and particularly the limitations section, is currently underdeveloped; the limitations paragraph addresses only the absence of career-level differentiation. A more comprehensive treatment would strengthen the manuscript and demonstrate balanced appraisal. The authors should expand the Discussion and limitations to address, among others, the occurrence of potential biases concerning the interviewing process (e.g. sampling bias, social desirability bias, interviewer biases) the low Kendall coefficient of concordance, the single-province sampling of the interviews, potential ceiling effects arising from the rating scale or the fact that the framework has not yet been empirically validated.

**Response:** Thank you for this comment. We have expanded the Discussion and Study Limitations to provide a more balanced appraisal of the study. The revised limitations now address the geographically concentrated sampling of the preliminary interviews, potential social desirability and interviewer influence, and the lack of empirical validation of the framework. The relatively low Kendall’s W values and potential ceiling effect are further discussed in the Discussion section. The revised text can be found in  **Lines 757-775 and Lines 572-581.**

**Minor issues/Minor comments**

1. Figure 1 — Typographical error. In the flowchart (Fig 1), "Delphi" is misspelled as "Dephi" in both "Dephi round 1" and "Dephi round 2." Please correct this.

**Response:** Thank you for pointing out this error. We apologize for this oversight. We have corrected “Dephi” to “Delphi” in both “Delphi round 1” and “Delphi round 2” in Figure 1. We have also carefully checked the figure for other typographical errors. The corrected Figure 1 has been provided in the revised manuscript.

2. Figure 1 - Image resolution. Figure 1 is difficult to read. Given the importance of the figure for conveying the overall study process, please also ensure it is supplied at sufficient resolution for publication.

**Response:** Thank you for this comment. Figure 1 has been revised to improve its readability and has been re-exported at a higher resolution in accordance with the PLOS ONE figure requirements. The revised Figure 1 has been provided with the revised manuscript.

3. Language and minor grammar issues. A few sentences would benefit from light editing for subject–verb agreement and clarity; for example, "the role of public health nurses (PHNs)…have become increasingly prominent" (lines 81–83) should read "has become." A general language revision is recommended.

**Response:** Thank you for pointing out this error, and we apologize for the oversight. The Introduction has since been substantially revised in response to other reviewer comments, and the sentence highlighted here has been removed. We have also carefully reviewed the entire manuscript and corrected grammatical and language issues.
