# Supplementary material for: Development of a competency framework for public health nurses in China: A modified Delphi study
Source: PLoS One. 2026 Oct 30;21(10):e0361940. doi: 10.1371/journal.pone.0361940 (PMC13722293; doi:10.1371/journal.pone.0361940)
Supplement: Supplementary file 13 [file pone.0361940.s016.docx]

**Response to Reviewers:**

**1. Definition of public health nurse**

The Introduction now states that public health nurse is not an official professional title in China and that the term refers to nurses in primary health care institutions who carry out public health nursing duties. The main responsibilities are also listed. This largely addresses my earlier concern. I suggest one additional sentence to clarify how this term relates to actual job titles in China, for example that such staff may be called community nurses, public health nursing post holders, or similar titles. This would help readers understand that public health nurse is an operational term rather than a formal title.

**Response:**Thank you for your helpful suggestion. We have added a sentence to the Introduction to clarify how the term “public health nurse” relates to the actual job titles and positions used in Chinese primary health care institutions. The added text can be found in **Lines 83-84.**

**2. Deepening the Discussion**

The response letter explains that international frameworks emphasize broad professional competencies, while the Chinese framework emphasizes frontline service delivery under the National BPHS, and that TCM health management is part of the BPHS. This explanation is still brief and does not fully explain why some competencies are emphasized and others receive lower weights. I suggest adding a subsection or several paragraphs on international comparison and policy and role differences, linking the weights to the policy system and the role boundaries of PHNs. For example:

(1) In this study, health education and health promotion received the highest weight (0.280); health management and community health surveillance and assessment both received 0.196; community public health emergency management, partnership development and maintenance, and professionalism and professional development each received 0.110. TCM health management received a relatively low subdomain weight of 0.081 and a global weight of 0.016; the subdomain mean was 4.48, and the means for its two competencies were 4.43 and 4.52. Note that 4.38 belongs to 3.4.2 Ability to manage health in workplace settings, not to TCM health management.

(2) International frameworks, such as those of the United States, Canada, and Japan, often emphasize policy and program planning, financial management, systems leadership, and communication and collaboration. In the Chinese framework, policy, financial management, and systems leadership are relatively weak or do not appear as separate competencies. This may reflect that PHNs in China currently focus mainly on implementing the standardized National BPHS rather than taking part in policy making, financial management, and systems leadership.

(3) Although TCM health management is part of the National BPHS, the Delphi experts gave it a relatively low subdomain weight. This may indicate that PHNs have limited autonomy in TCM health management and mainly assist, refer, or provide health education rather than independently providing core TCM diagnosis and treatment. The authors should explain this explicitly in the Discussion. Otherwise, readers may ask why TCM health management is assigned a relatively low subdomain weight despite being a BPHS item.

**Response:**

Thank you for this helpful suggestion. We have expanded the Discussion to better explain the weighting results and international differences in relation to China’s policy context and the professional responsibilities of PHNs.

First, we have strengthened the explanation for the highest weight assigned to health education and health promotion by highlighting its role across BPHS services and its alignment with routine frontline responsibilities. The revised text can be found in **Lines 527-532**.

Second, we have clarified that the relatively low weight of TCM health management does not imply low perceived importance. We also explain how service-specific qualification requirements and professional role boundaries may partly account for this finding. The revised text can be found in **Lines 552-565**.

Regarding item 3.4.2, “Ability to manage health in workplace settings,” we would like to clarify the relationship between importance ratings and global weights. When comparing the global weights of third-level competencies across different second-level subdomains, both their local weights and their parent subdomain weights must be considered. Workplace health management and knowledge of TCM health management both have a local weight of 0.333, but their parent subdomains have weights of 0.137 and 0.081, respectively, resulting in global weights of 0.009 and 0.005. Thus, the lower importance rating for workplace health management does not conflict with its higher global weight in this comparison.

Third, we have expanded the international comparison to explain how the framework’s frontline BPHS orientation may account for the absence of separate policy development, financial management, and systems leadership domains. We also highlight the need to further define these competency requirements for different professional roles and career stages. The revised text can be found in **Lines 639-648.**

**3. Inconsistent terminology**

The revised manuscript still uses inconsistent terminology. Reviewer #2 asked for consistent use of domains, subdomains, and competencies, but this has not been fully implemented. For example, the Abstract contains the following formulations:

(1) six domains, 16 subdomains, and 52 competencies

(2) six primary domains, 16 secondary indicators, and 52 tertiary indicators

(3) six primary, 16 secondary, and 46 tertiary indicators

**Response:** Thank you for emphasizing the need for consistent terminology. We have rechecked the entire manuscript and confirmed that “domains,” “subdomains,” and “competencies” are consistently used to describe the three hierarchical levels.
