# Supplementary material for: Development of a competency framework for public health nurses in China: A modified Delphi study
Source: PLoS One. 2026 Oct 30;21(10):e0361940. doi: 10.1371/journal.pone.0361940 (PMC13722293; doi:10.1371/journal.pone.0361940)
Supplement: Peer review history

# Peer review: Decision Letter 0

**DOI:** 10.1371/journal.pone.0361940.r001

**Authors:** Mohd Ismail Ibrahim

16 Jun 2026

Dear Dr. Zhang,

Thank you for submitting your manuscript to PLOS ONE. After careful consideration, we feel that it has merit but does not fully meet PLOS ONE’s publication criteria as it currently stands. Therefore, we invite you to submit a revised version of the manuscript that addresses the points raised during the review process.

Please submit your revised manuscript by Jul 31 2026 11:59PM. If you will need more time than this to complete your revisions, please reply to this message or contact the journal office at plosone@plos.org. When you're ready to submit your revision, log on to https://www.editorialmanager.com/pone/ and select the 'Submissions Needing Revision' folder to locate your manuscript file.

- A letter that responds to each point raised by the academic editor and reviewer(s). You should upload this letter as a separate file labeled 'Response to Reviewers'.
- A marked-up copy of your manuscript that highlights changes made to the original version. You should upload this as a separate file labeled 'Revised Manuscript with Track Changes'.
- An unmarked version of your revised paper without tracked changes. You should upload this as a separate file labeled 'Manuscript'.

If you would like to make changes to your financial disclosure, please include your updated statement in your cover letter. Guidelines for resubmitting your figure files are available below the reviewer comments at the end of this letter.

If applicable, we recommend that you deposit your laboratory protocols in protocols.io to enhance the reproducibility of your results. Protocols.io assigns your protocol its own identifier (DOI) so that it can be cited independently in the future. For instructions see: https://journals.plos.org/plosone/s/submission-guidelines#loc-laboratory-protocols. Additionally, PLOS ONE offers an option for publishing peer-reviewed Lab Protocol articles, which describe protocols hosted on protocols.io. Read more information on sharing protocols at https://plos.org/protocols?utm_medium=editorial-email&utm_source=authorletters&utm_campaign=protocols.

As the corresponding author, your ORCID iD is verified in the submission system and will appear in the published article. PLOS supports the use of ORCID, and we encourage all coauthors to register for an ORCID iD and use it as well. Please encourage your coauthors to verify their ORCID iD within the submission system before final acceptance, as unverified ORCID iDs will not appear in the published article. *Only*  the individual author can complete the verification step; PLOS staff *cannot*  verify ORCID iDs on behalf of authors.

We look forward to receiving your revised manuscript.

Kind regards,

Mohd Ismail Ibrahim, MCom.Med

Academic Editor

PLOS One

Journal requirements:

When submitting your revision, we need you to address these additional requirements.

1. Please ensure that your manuscript meets PLOS ONE's style requirements, including those for file naming. The PLOS ONE style templates can be found at

https://journals.plos.org/plosone/s/file?id=wjVg/PLOSOne_formatting_sample_main_body.pdf and

https://journals.plos.org/plosone/s/file?id=ba62/PLOSOne_formatting_sample_title_authors_affiliations.pdf

2. When completing the data availability statement of the submission form, you indicated that you will make your data available on acceptance. We strongly recommend all authors decide on a data sharing plan before acceptance, as the process can be lengthy and hold up publication timelines. Please note that, though access restrictions are acceptable now, your entire data will need to be made freely accessible if your manuscript is accepted for publication. This policy applies to all data except where public deposition would breach compliance with the protocol approved by your research ethics board. If you are unable to adhere to our open data policy, please kindly revise your statement to explain your reasoning and we will seek the editor's input on an exemption. Please be assured that, once you have provided your new statement, the assessment of your exemption will not hold up the peer review process.

If the reviewer comments include a recommendation to cite specific previously published works, please review and evaluate these publications to determine whether they are relevant and should be cited. There is no requirement to cite these works unless the editor has indicated otherwise.

[Note: HTML markup is below. Please do not edit.]

Reviewers' comments:

Reviewer's Responses to Questions

**Comments to the Author**

1. Is the manuscript technically sound, and do the data support the conclusions?

Reviewer #1: Yes

Reviewer #2: Partly

Reviewer #3: Yes

**********

2. Has the statistical analysis been performed appropriately and rigorously?

Reviewer #1: Yes

Reviewer #2: I Don't Know

Reviewer #3: Yes

**********

3. Have the authors made all data underlying the findings in their manuscript fully available?

The PLOS Data policy

Reviewer #1: No

Reviewer #2: No

Reviewer #3: Yes

**********

4. Is the manuscript presented in an intelligible fashion and written in standard English?

Reviewer #1: Yes

Reviewer #2: Yes

Reviewer #3: Yes

**********

Reviewer #1: Dear Authors,

Thank you for the opportunity to review your work, a Delphi study aimed at developing a competency framework for public health nurses in China. I have read your paper with great care and interest. I have the following suggestions:

MAJOR COMMENTS

1. the competency framework is structured into three levels (domains, sub-domains, and tertiary indicators). The third-level indicators are detailed in the Supplementary Materials. However, a major area for methodological improvement is that these third-level descriptions are predominantly descriptive and task-oriented, lacking a rigorous taxonomic structure. I fully recognize that the current wording is the direct outcome of the Delphi consensus. Consequently, the authors cannot arbitrarily rewrite these items using taxonomic verbs without invalidating the study results. To reconcile the finalized Delphi consensus with the need for educational and taxonomic rigor, I suggest addressing this limitation through one or both of the following actions:

(a) add supplementary columns to the framework that retroactively classify each item by its conceptual domain and map it to a recognized taxonomic framework (e.g., Bloom's taxonomy) to act as an interpretative layer without altering the original text and the study findings;

(b) explicitly address this constraint in the Limitations section, underlining that translating these functional tasks into pedagogically measurable competencies, with clearly defined levels of nursing autonomy, will be a fundamental and necessary step for future research.

MINOR COMMENTS

2. the first opening paragraph of the Introduction seems a bit broad and not perfectly aligned with the study's objectives. While citing these global issues creates a sense of urgency, these concepts are not well integrated with the rest of the text. I suggest rewriting this first paragraph to be more brief.

3. on page 8, the relation between community nursing and public health nursing is introduced, however a specific supporting reference is missing;

4. in the Methods section, the authors mention the use of "maximum variation purposive sampling". I suggest adding a brief rationale and a supporting methodological reference;

5. in the demographic tables, mirroring data (e.g., male/female) is redundant. I suggest collapsing these variables to improve readability;

6. according to what was introduced in the background, the division of the practitioners' field of expertise into "community nursing", "public health", and "health education" appears unclear. Given that these fields heavily overlap (as noted in the Background), I suggest reformulating and restructuring this categorization to avoid taxonomic ambiguity or providing a rationale;

7. the text cites the high response rate as evidence of the Delphi process's quality. However, with a purposively selected sample of 22 subjects, the response rate should not be presented as the primary indicator of strength. I suggest shifting the emphasis away from the sample size and instead highlighting the high quality, expertise, and specific competencies of the professionals involved in the panel.

I hope that my suggestions will be helpful in improving your work.

Reviewer #2: Please find attached the report with review comments to the Authors.

Please find attached the report with review comments to the Authors.

Please find attached the report with review comments to the Authors.

Reviewer #3: 1. Clearly define the concept of "public health nurse"

The term "public health nurse" is not commonly used in China's healthcare setting. Although the authors mention the relationship between public health nursing and community nursing in the introduction, this relationship is still not very clear to readers. I suggest that the authors provide a clearer definition of "public health nurse" in the introduction section, explaining how this role is understood and positioned within China's current health system.

2. Limitations regarding the representativeness of the expert panel

2.1. Geographic imbalance: Among the 21 experts, 52.4% came from Western China. Given the notable differences in healthcare development levels between Eastern and Western China, the results may be biased toward experiences from less developed regions, and the framework's applicability to more developed Eastern regions remains uncertain. The authors should mention this in the limitations section.

2.2. Professional background imbalance: Most of the authors' experts (57.1%) had a background in community nursing, while only 14.3% had a background in public health. The competency framework the authors developed is for public health nurses, so would it not be more appropriate to have a stronger public health perspective rather than a nursing perspective? I suggest that the authors acknowledge this bias in the limitations and note that future studies should include more experts from public health, health management, epidemiology, and other related fields.

3. Deepen the discussion section

The authors compared their framework with those from the United States, Canada, and Japan, but the comparison mainly stays at the level of "similarities". I think the authors need to go deeper into why certain competencies are emphasized in China (such as 3.5 TCM health management competency) while others are given less weight. A discussion of how differences in policy systems or nursing role definitions across countries might explain these differences would strengthen this section.

4. Conclusion needs clearer guidance for next steps

The conclusion states that the framework demonstrates methodological rigor and contextual relevance. I suggest a more cautious tone, clearly stating that this framework has not yet been empirically validated. The authors should specify what should be done next, for example: testing the reliability and validity of the framework, pilot applications in real settings, and developing tiered competency standards for different career levels.

**********

what does this mean?). If published, this will include your full peer review and any attached files.

If you choose “no”, your identity will remain anonymous but your review may still be made public.

**Do you want your identity to be public for this peer review?**  For information about this choice, including consent withdrawal, please see our Privacy Policy

Reviewer #1: No

Reviewer #2: No

Reviewer #3: **Yes:**  He Jiang

**********

[NOTE: If reviewer comments were submitted as an attachment file, they will be attached to this email and accessible via the submission site. Please log into your account, locate the manuscript record, and check for the action link "View Attachments". If this link does not appear, there are no attachment files.]

To ensure your figures meet our technical requirements, please review our figure guidelines: https://journals.plos.org/plosone/s/figures

You may also use PLOS’s free figure tool, NAAS, to help you prepare publication quality figures: https://journals.plos.org/plosone/s/figures#loc-tools-for-figure-preparation.

NAAS will assess whether your figures meet our technical requirements by comparing each figure against our figure specifications.

Attachment Submitted filename: Review manuscript PONE-D-26-02847_Comments to Authors.pdf

Attachment Submitted filename: Confidential Comments to the Editor PONE-D-26-02847.pdf

# Author comment: Author response to Decision Letter 1

**DOI:** 10.1371/journal.pone.0361940.r002

31 Aug 2026

We sincerely thank the Academic Editor and the reviewers for their thoughtful and constructive comments. We have carefully addressed all comments and revised the manuscript accordingly. Detailed point-by-point responses to all comments are provided in the separately uploaded “Response to Reviewers” file.

Attachment Submitted filename: Response to Reviewers.docx

# Peer review: Decision Letter 1

**DOI:** 10.1371/journal.pone.0361940.r003

**Authors:** Mohd Ismail Ibrahim

5 Oct 2026

Dear Dr. Zhang,

Thank you for submitting your manuscript to PLOS One. After careful consideration, we feel that it has merit but does not fully meet PLOS One’s publication criteria as it currently stands. Therefore, we invite you to submit a revised version of the manuscript that addresses the points raised during the review process.

Please submit your revised manuscript by Nov 19 2026 11:59PM. If you will need more time than this to complete your revisions, please reply to this message or contact the journal office at plosone@plos.org. When you're ready to submit your revision, log on to https://www.editorialmanager.com/pone/ and select the 'Submissions Needing Revision' folder to locate your manuscript file.

- A letter that responds to each point raised by the academic editor and reviewer(s). You should upload this letter as a separate file labeled 'Response to Reviewers'.
- A marked-up copy of your manuscript that highlights changes made to the original version. You should upload this as a separate file labeled 'Revised Manuscript with Track Changes'.
- An unmarked version of your revised paper without tracked changes. You should upload this as a separate file labeled 'Manuscript'.

If you would like to make changes to your financial disclosure, please include your updated statement in your cover letter. Guidelines for resubmitting your figure files are available below the reviewer comments at the end of this letter.

If applicable, we recommend that you deposit your laboratory protocols in protocols.io to enhance the reproducibility of your results. Protocols.io assigns your protocol its own identifier (DOI) so that it can be cited independently in the future. For instructions see: https://journals.plos.org/plosone/s/submission-guidelines#loc-laboratory-protocols. Additionally, PLOS One offers an option for publishing peer-reviewed Lab Protocol articles, which describe protocols hosted on protocols.io. Read more information on sharing protocols at https://plos.org/protocols?utm_medium=editorial-email&utm_source=authorletters&utm_campaign=protocols.

As the corresponding author, your ORCID iD is verified in the submission system and will appear in the published article. PLOS supports the use of ORCID, and we encourage all coauthors to register for an ORCID iD and use it as well. Please encourage your coauthors to verify their ORCID iD within the submission system before final acceptance, as unverified ORCID iDs will not appear in the published article. *Only*  the individual author can complete the verification step; PLOS staff *cannot*  verify ORCID iDs on behalf of authors.

We look forward to receiving your revised manuscript.

Kind regards,

Mohd Ismail Ibrahim, MCom.Med

Academic Editor

PLOS One

Journal Requirements:

1. If the reviewer comments include a recommendation to cite specific previously published works, please review and evaluate these publications to determine whether they are relevant and should be cited. There is no requirement to cite these works unless the editor has indicated otherwise.

2. Please review your reference list to ensure that it is complete and correct. If you have cited papers that have been retracted, please include the rationale for doing so in the manuscript text, or remove these references and replace them with relevant current references. Any changes to the reference list should be mentioned in the rebuttal letter that accompanies your revised manuscript. If you need to cite a retracted article, indicate the article’s retracted status in the References list and also include a citation and full reference for the retraction notice.

[Note: HTML markup is below. Please do not edit.]

Reviewers' comments:

Reviewer's Responses to Questions

**Comments to the Author**

Reviewer #1: All comments have been addressed

Reviewer #3: (No Response)

**********

2. Is the manuscript technically sound, and do the data support the conclusions?

Reviewer #1: Yes

Reviewer #3: Yes

**********

3. Has the statistical analysis been performed appropriately and rigorously?

Reviewer #1: N/A

Reviewer #3: Yes

**********

4. Have the authors made all data underlying the findings in their manuscript fully available?

The PLOS Data policy

Reviewer #1: Yes

Reviewer #3: Yes

**********

5. Is the manuscript presented in an intelligible fashion and written in standard English?

Reviewer #1: Yes

Reviewer #3: Yes

**********

Reviewer #1: All my previous suggestions have been adequately addressed and I have no further comments. Thank you.

Reviewer #3: The authors have responded thoroughly to the previous comments. The manuscript is much improved, and most major concerns have been addressed. Some points still need attention.

1. Definition of public health nurse

The Introduction now states that public health nurse is not an official professional title in China and that the term refers to nurses in primary health care institutions who carry out public health nursing duties. The main responsibilities are also listed. This largely addresses my earlier concern. I suggest one additional sentence to clarify how this term relates to actual job titles in China, for example that such staff may be called community nurses, public health nursing post holders, or similar titles. This would help readers understand that public health nurse is an operational term rather than a formal title.

2. Deepening the Discussion

The response letter explains that international frameworks emphasize broad professional competencies, while the Chinese framework emphasizes frontline service delivery under the National BPHS, and that TCM health management is part of the BPHS. This explanation is still brief and does not fully explain why some competencies are emphasized and others receive lower weights. I suggest adding a subsection or several paragraphs on international comparison and policy and role differences, linking the weights to the policy system and the role boundaries of PHNs. For example:

(1) In this study, health education and health promotion received the highest weight (0.280); health management and community health surveillance and assessment both received 0.196; community public health emergency management, partnership development and maintenance, and professionalism and professional development each received 0.110. TCM health management received a relatively low subdomain weight of 0.081 and a global weight of 0.016; the subdomain mean was 4.48, and the means for its two competencies were 4.43 and 4.52. Note that 4.38 belongs to 3.4.2 Ability to manage health in workplace settings, not to TCM health management.

(2) International frameworks, such as those of the United States, Canada, and Japan, often emphasize policy and program planning, financial management, systems leadership, and communication and collaboration. In the Chinese framework, policy, financial management, and systems leadership are relatively weak or do not appear as separate competencies. This may reflect that PHNs in China currently focus mainly on implementing the standardized National BPHS rather than taking part in policy making, financial management, and systems leadership.

(3) Although TCM health management is part of the National BPHS, the Delphi experts gave it a relatively low subdomain weight. This may indicate that PHNs have limited autonomy in TCM health management and mainly assist, refer, or provide health education rather than independently providing core TCM diagnosis and treatment. The authors should explain this explicitly in the Discussion. Otherwise, readers may ask why TCM health management is assigned a relatively low subdomain weight despite being a BPHS item.

3. Inconsistent terminology

The revised manuscript still uses inconsistent terminology. Reviewer #2 asked for consistent use of domains, subdomains, and competencies, but this has not been fully implemented. For example, the Abstract contains the following formulations:

(1) six domains, 16 subdomains, and 52 competencies

(2) six primary domains, 16 secondary indicators, and 52 tertiary indicators

(3) six primary, 16 secondary, and 46 tertiary indicators

Recommendation: minor revision.

**********

what does this mean?). If published, this will include your full peer review and any attached files.

If you choose “no”, your identity will remain anonymous but your review may still be made public.

**Do you want your identity to be public for this peer review?**  For information about this choice, including consent withdrawal, please see our Privacy Policy

Reviewer #1: No

Reviewer #3: No

**********

[NOTE: If reviewer comments were submitted as an attachment file, they will be attached to this email and accessible via the submission site. Please log into your account, locate the manuscript record, and check for the action link "View Attachments". If this link does not appear, there are no attachment files.]

To ensure your figures meet our technical requirements, please review our figure guidelines: https://journals.plos.org/plosone/s/figures

You may also use PLOS’s free figure tool, NAAS, to help you prepare publication quality figures: https://journals.plos.org/plosone/s/figures#loc-tools-for-figure-preparation.

NAAS will assess whether your figures meet our technical requirements by comparing each figure against our figure specifications.

Attachment Submitted filename: renamed_e5cf4.doc

# Author comment: Author response to Decision Letter 2

**DOI:** 10.1371/journal.pone.0361940.r004

10 Oct 2026

Response to Reviewers:

1. Definition of public health nurse

The Introduction now states that public health nurse is not an official professional title in China and that the term refers to nurses in primary health care institutions who carry out public health nursing duties. The main responsibilities are also listed. This largely addresses my earlier concern. I suggest one additional sentence to clarify how this term relates to actual job titles in China, for example that such staff may be called community nurses, public health nursing post holders, or similar titles. This would help readers understand that public health nurse is an operational term rather than a formal title.

Response:Thank you for your helpful suggestion. We have added a sentence to the Introduction to clarify how the term “public health nurse” relates to the actual job titles and positions used in Chinese primary health care institutions. The added text can be found in Lines 83-84.

2. Deepening the Discussion

The response letter explains that international frameworks emphasize broad professional competencies, while the Chinese framework emphasizes frontline service delivery under the National BPHS, and that TCM health management is part of the BPHS. This explanation is still brief and does not fully explain why some competencies are emphasized and others receive lower weights. I suggest adding a subsection or several paragraphs on international comparison and policy and role differences, linking the weights to the policy system and the role boundaries of PHNs. For example:

(1) In this study, health education and health promotion received the highest weight (0.280); health management and community health surveillance and assessment both received 0.196; community public health emergency management, partnership development and maintenance, and professionalism and professional development each received 0.110. TCM health management received a relatively low subdomain weight of 0.081 and a global weight of 0.016; the subdomain mean was 4.48, and the means for its two competencies were 4.43 and 4.52. Note that 4.38 belongs to 3.4.2 Ability to manage health in workplace settings, not to TCM health management.

(2) International frameworks, such as those of the United States, Canada, and Japan, often emphasize policy and program planning, financial management, systems leadership, and communication and collaboration. In the Chinese framework, policy, financial management, and systems leadership are relatively weak or do not appear as separate competencies. This may reflect that PHNs in China currently focus mainly on implementing the standardized National BPHS rather than taking part in policy making, financial management, and systems leadership.

(3) Although TCM health management is part of the National BPHS, the Delphi experts gave it a relatively low subdomain weight. This may indicate that PHNs have limited autonomy in TCM health management and mainly assist, refer, or provide health education rather than independently providing core TCM diagnosis and treatment. The authors should explain this explicitly in the Discussion. Otherwise, readers may ask why TCM health management is assigned a relatively low subdomain weight despite being a BPHS item.

Response:

Thank you for this helpful suggestion. We have expanded the Discussion to better explain the weighting results and international differences in relation to China’s policy context and the professional responsibilities of PHNs.

First, we have strengthened the explanation for the highest weight assigned to health education and health promotion by highlighting its role across BPHS services and its alignment with routine frontline responsibilities. The revised text can be found in Lines 527-532.

Second, we have clarified that the relatively low weight of TCM health management does not imply low perceived importance. We also explain how service-specific qualification requirements and professional role boundaries may partly account for this finding. The revised text can be found in Lines 552-565.

Regarding item 3.4.2, “Ability to manage health in workplace settings,” we would like to clarify the relationship between importance ratings and global weights. When comparing the global weights of third-level competencies across different second-level subdomains, both their local weights and their parent subdomain weights must be considered. Workplace health management and knowledge of TCM health management both have a local weight of 0.333, but their parent subdomains have weights of 0.137 and 0.081, respectively, resulting in global weights of 0.009 and 0.005. Thus, the lower importance rating for workplace health management does not conflict with its higher global weight in this comparison.

Third, we have expanded the international comparison to explain how the framework’s frontline BPHS orientation may account for the absence of separate policy development, financial management, and systems leadership domains. We also highlight the need to further define these competency requirements for different professional roles and career stages. The revised text can be found in Lines 639-648.

3. Inconsistent terminology

The revised manuscript still uses inconsistent terminology. Reviewer #2 asked for consistent use of domains, subdomains, and competencies, but this has not been fully implemented. For example, the Abstract contains the following formulations:

(1) six domains, 16 subdomains, and 52 competencies

(2) six primary domains, 16 secondary indicators, and 52 tertiary indicators

(3) six primary, 16 secondary, and 46 tertiary indicators

Response: Thank you for emphasizing the need for consistent terminology. We have rechecked the entire manuscript and confirmed that “domains,” “subdomains,” and “competencies” are consistently used to describe the three hierarchical levels.

Attachment Submitted filename: Response_to_Reviewers_auresp_2.docx

# Peer review: Decision Letter 2

**DOI:** 10.1371/journal.pone.0361940.r005

**Authors:** Mohd Ismail Ibrahim

19 Oct 2026

Development of a competency framework for public health nurses in China: A modified Delphi study

PONE-D-26-02847R2

Dear Dr. Zhang,

We’re pleased to inform you that your manuscript has been judged scientifically suitable for publication and will be formally accepted for publication once it meets all outstanding technical requirements.

Within one week, you’ll receive an e-mail detailing the required amendments. When these have been addressed, you’ll receive a formal acceptance letter and your manuscript will be scheduled for publication.

An invoice will be generated when your article is formally accepted. Please note, if your institution has a publishing partnership with PLOS and your article meets the relevant criteria, all or part of your publication costs will be covered. Please make sure your user information is up-to-date by logging into Editorial Manager at Editorial Manager® and clicking the ‘Update My Information' link at the top of the page. For questions related to billing, please contact billing support.

If your institution or institutions have a press office, please notify them about your upcoming paper to help maximize its impact. If they’ll be preparing press materials, please inform our press team as soon as possible -- no later than 48 hours after receiving the formal acceptance. Your manuscript will remain under strict press embargo until 2 pm Eastern Time on the date of publication. For more information, please contact onepress@plos.org.

Kind regards,

Mohd Ismail Ibrahim, MCom.Med

Academic Editor

PLOS One

Additional Editor Comments (optional):

The authors have satisfactorily addressed the concerns raised during the peer review process. The revised manuscript is methodologically sound, clearly presented, and provides a valuable contribution to public health nursing competency development. I agree with the reviewer's recommendation and consider the manuscript suitable for publication in its current form. Therefore, I recommend acceptance for publication.

Reviewers' comments:

Reviewer's Responses to Questions

**Comments to the Author**

Reviewer #3: All comments have been addressed

**********

2. Is the manuscript technically sound, and do the data support the conclusions?

Reviewer #3: Yes

**********

3. Has the statistical analysis been performed appropriately and rigorously?

Reviewer #3: Yes

**********

4. Have the authors made all data underlying the findings in their manuscript fully available?

The PLOS Data policy

Reviewer #3: Yes

**********

5. Is the manuscript presented in an intelligible fashion and written in standard English?

Reviewer #3: Yes

**********

Reviewer #3: All my prior suggestions have been satisfactorily addressed, I have no additional comments. Thank you.

**********

what does this mean?). If published, this will include your full peer review and any attached files.

If you choose “no”, your identity will remain anonymous but your review may still be made public.

**Do you want your identity to be public for this peer review?**  For information about this choice, including consent withdrawal, please see our Privacy Policy

Reviewer #3: No

**********

# Editor report: Acceptance letter

**DOI:** 10.1371/journal.pone.0361940.r006

**Authors:** Mohd Ismail Ibrahim

PONE-D-26-02847R2

PLOS One

Dear Dr. Zhang,

I'm pleased to inform you that your manuscript has been deemed suitable for publication in PLOS One. Congratulations! Your manuscript is now being handed over to our production team.

At this stage, our production department will prepare your paper for publication. This includes ensuring the following:

* All references, tables, and figures are properly cited

* All relevant supporting information is included in the manuscript submission,

* There are no issues that prevent the paper from being properly typeset

You will receive further instructions from the production team, including instructions on how to review your proof when it is ready. Please keep in mind that we are working through a large volume of accepted articles, so please give us a few days to review your paper and let you know the next and final steps.

Lastly, if your institution or institutions have a press office, please let them know about your upcoming paper now to help maximize its impact. If they'll be preparing press materials, please inform our press team within the next 48 hours. Your manuscript will remain under strict press embargo until 2 pm Eastern Time on the date of publication. For more information, please contact onepress@plos.org.

You will receive an invoice from PLOS for your publication fee after your manuscript has reached the completed accept phase. If you receive an email requesting payment before acceptance or for any other service, this may be a phishing scheme. Learn how to identify phishing emails and protect your accounts at https://explore.plos.org/phishing.

If we can help with anything else, please email us at customercare@plos.org.

Thank you for submitting your work to PLOS One and supporting open access.

Kind regards,

PLOS One Editorial Office Staff

on behalf of

Dr. Mohd Ismail Ibrahim

Academic Editor

PLOS One
